# FISH-Dist: An Automated Pipeline for 3D Genomic Spatial Distance Quantification in FISH Imaging

**DOI:** 10.3390/bioengineering13030268

**Published:** 2026-02-26

**Authors:** Benoit Aigouy, Emmanuelle Caturegli, Bernard Charroux, Carla Silva Martins, Thomas Gregor, Benjamin Prud’homme

**Affiliations:** 1 Aix-Marseille Université, CNRS, IBDM, Campus de Luminy Case 907, 13288 Marseille Cedex 9, France; emmanuelle.caturegli@univ-amu.fr (E.C.); bernard.charroux@univ-amu.fr (B.C.);; 2 Lewis–Sigler Institute for Integrative Genomics and Joseph Henry Laboratories of Physics, Princeton University, Princeton, NJ 08544, USA; 3 Department of Developmental and Stem Cell Biology, CNRS UMR3738 Paris Cité, Institut Pasteur, 75015 Paris, France

**Keywords:** fluorescence in situ hybridization (FISH), Oligopaint probes, chromatic aberration correction, 3D genome organization, short-range genomic interactions, distance quantification, sub-pixel localization, confocal microscopy, computational image analysis

## Abstract

Accurate quantification of spatial distances between fluorescent signals in multi-channel 3D microscopy is essential for understanding genomic organization and gene regulation. However, chromatic aberration introduces systematic spatial offsets between channels that significantly bias distance measurements, particularly at short genomic distances. We present FISH-Dist, an automated computational pipeline for quantitative distance measurements in 3D fluorescence in situ hybridization (FISH) experiments acquired on standard confocal microscopes. Our method combines deep learning-based spot segmentation, 3D Gaussian fitting for sub-pixel localization, and two complementary chromatic aberration correction approaches: affine (ACC) and linear (LCC). We validated the pipeline by measuring the lengths of DNA origami nanorulers and systematically evaluated FISH probe design parameters, including probe spacing, density, and target sequence length. FISH-Dist achieves sub-pixel accuracy in signal detection and substantially reduces inter-channel distance measurement errors. This enables a reproducible quantification of spatial relationships in 3D FISH datasets. Unlike existing tools optimized for long-range chromosomal interactions or requiring super-resolution microscopy, FISH-Dist specifically addresses the technical challenges of standard confocal imaging at short genomic distances, where chromatic aberration has a proportionally greater impact on measurement accuracy.

## 1. Introduction

The three-dimensional organization of the genome is fundamental to gene regulation [1], as spatial proximity between regulatory elements and their target genes directly influences transcription [2,3]. Fluorescence in situ hybridization (FISH), including modern Oligopaint-based approaches, remains a gold-standard method for visualizing these spatial relationships with single-cell resolution within the native nuclear context [4,5,6,7].

While existing FISH studies and computational tools, sometimes complemented by Hi-C-based maps, have significantly advanced our understanding of large-scale chromosomal structures and long-range interactions spanning hundreds of kilobases to megabases [6,8,9,10,11,12,13], many critical regulatory interactions—such as enhancer–promoter loops and local chromatin contacts—occur at much finer genomic scales (<10–100 kb) [14]. At these scales, technical limitations, including chromatic aberration, can introduce measurement errors that are comparable to, or even exceed, the biological distances being investigated.

Despite the biological significance of short-range genomic measurements, many studies still rely on user-dependent distance measurements, which are prone to error [15,16,17,18]. Existing computational tools are typically not designed or rigorously validated for high-precision quantification at these scales. Moreover, the relationship between FISH probe design parameters—such as probe density and target sequence length—and measurement accuracy remains poorly characterized for short-distance applications.

Here we present FISH-Dist, an automated computational pipeline for quantitative 3D distance measurements in FISH imaging, with a focus on short-range genomic distances. FISH-Dist integrates deep learning-based spot detection [19], sub-pixel 3D Gaussian fitting, and complementary chromatic aberration correction strategies for high-resolution measurements. We validate the pipeline using orthogonal approaches, including colocalization experiments and calibrated nanorulers. Using FISH-Dist, we also systematically quantify how probe design parameters, such as labeling density and target sequence length, influence measurement accuracy, providing practical guidance for experimental planning. By addressing technical limitations in standard confocal FISH, FISH-Dist provides a reproducible framework for quantifying spatial relationships at the short genomic distances most relevant to gene regulation.

## 2. Materials and Methods

### 2.1. Oligopaint Probe Synthesis

Forty-five-nucleotide single-stranded DNA Oligopaint probes targeting specific DNA sequences were designed by Daicel Arbor Biosciences and synthesized from myTags Immortal Libraries (Daicel Arbor Biosciences, Ann Arbor, MI, USA) according to the manufacturer’s protocol [20]. Briefly, myTags Immortal Libraries were amplified by PCR, and the resulting products were column-purified. In vitro transcription was then performed to generate RNA, which was column-purified and used as a template for reverse transcription (RT). When RNA concentration was insufficient for RT, an additional ethanol precipitation was performed. RT was carried out using fluorescent primers (coupled at the 5′ end with a single ATTO 565 or ATTO 633 fluorescent dye) purchased from Integrated DNA Technologies (Coralville, IA, USA). Unincorporated primers were degraded, and RNA:DNA hybrids were column-purified. RNA fragments were subsequently removed by RNAse treatment, and the resulting fluorescent single-stranded DNA probes were column-purified and stored at −20 °C. The fraction of successfully dye-conjugated probes was quantified using a Nanodrop spectrophotometer (Thermo Fisher Scientific, Waltham, MA, USA) to ensure an appropriate labeling ratio. Detailed sequences for all oligos are available in Supplementary File S1.

### 2.2. Oligopaint Probe Design for Spatial Resolution Calibration in Multi-Color FISH

Oligopaint probes were designed to target a ∼10 kb endogenous genomic region (X:6,760,094–6,770,369, Drosophila genome Release 6) adjacent to the attP18 landing site on the X chromosome of *Drosophila melanogaster*. A total of 142 Oligopaint probes were designed to hybridize exclusively to the + DNA strand of this locus. For dual-color experiments, the same probe set was labeled with either ATTO 565 or ATTO 633 fluorophores.

### 2.3. Construction and Oligopaint Targeting of Synthetic Reporter Sequences

Two synthetic reporter sequences (R1 and R6) were designed to be orthogonal to the *Drosophila melanogaster* genome by Daicel Arbor Biosciences and synthesized by Genewiz. Each reporter consists of a 2 kb DNA sequence absent from the *D. melanogaster* genome, with similar overall GC content but distinct sequence composition. Each reporter was targeted by a set of 87 Oligopaint probes tiled across the entire sequence and both DNA strands. Transgenes were integrated at the ZH-2A landing site on the X chromosome using site-specific recombination. Detailed sequences for all oligos are available in Supplementary File S1.

### 2.4. Transgene Design for Defined 3D Genomic Distance Measurements

To evaluate the accuracy of 3D distance measurements under biological conditions, we generated transgenic constructs containing two distinct Oligopaint-tagged sets targeting R1 and R6, labeled with ATTO 565 and ATTO 633 and separated by defined spacer lengths of 0 kb, 3 kb, or 10 kb. In all constructs, the spacer is flanked on both sides by the synthetic reporter tags R1 and R6, which serve as independent Oligopaint target sites. 3kb spacer consists of randomly generated DNA sequences designed to minimize binding of known *Drosophila* transcription factors. The 10 kb spacer is a cherry-tagged *yellow* gene of *Drosophila biarmipes*. All constructs were integrated at the ZH-2A landing site. Detailed sequences for all constructs are available in Supplementary File S1.

### 2.5. Oligopaint Target Length Variants for Assessing Spatial Resolution

To examine how FISH target sequence length influences spatial resolution, a series of transgenic constructs was generated in which progressively shorter segments of the R6 synthetic reporter were used as Oligopaint targets. Three target lengths were tested—2 kb, 1 kb, and 500 bp—preserving sequence composition while varying target size. All constructs were integrated at the ZH-2A landing site, ensuring a constant genomic context across conditions.

### 2.6. Modulation of Oligopaint Probe Density

To assess the impact of Oligopaint probe density on measurement performance, the fraction of fluorescently labeled probes targeting a fixed 2 kb R6 genomic region was systematically varied while maintaining a constant total probe concentration. This was achieved by mixing fluorescently labeled probes with unlabeled (“cold”) probes targeting the same sequence, thereby preserving hybridization conditions while progressively reducing the number of fluorescent probes contributing to the detected signal.

Three labeling conditions were examined. In the fully labeled condition (∼100%), the 2 kb R6 locus could be covered by up to 87 labeled probes and no unlabeled probes; ATTO 565- and ATTO 633-labeled probe sets were each used at a final concentration of 3 ng/µL, resulting in a total labeled probe concentration of 6 ng/µL. In the intermediate labeling condition (∼66%), the locus could be covered by up to 57 labeled probes and 30 unlabeled probes; each labeled probe set was used at 2 ng/µL and supplemented with 2 ng/µL of unlabeled probes. In the lowest labeling condition (∼50%), the locus could be covered by up to 43 labeled probes and 43 unlabeled probes; here, each labeled probe set was used at 1.5 ng/µL and supplemented with 3 ng/µL of unlabeled probes. In all cases, the total probe concentration was maintained at 6 ng/µL. Hybridization, imaging, and signal detection were performed under standard conditions.

### 2.7. Fluorescence In Situ Hybridization on Late Pupal Wings

At 70 h after puparium formation (APF), male flies -carrying a single X chromosome, and therefore a single set of each probe- were immobilized on adhesive tape, removed from their pupal cases, and gently dragged across the tape surface to remove residual cuticle shed during pupal development. Specimens were transferred to a small dish containing deionized water for approximately 1 min to allow wing unfolding. Wings were fixed in 4% formaldehyde in PBTT (1× PBS, 0.1% Tween-20, 0.3% Triton X-100) for 30 min at room temperature (RT), then washed twice in PBS. At 70 h APF, wings are enclosed by non-permeable cuticle and therefore require mechanical separation of the dorsal and ventral epithelial layers prior to probe hybridization. To achieve this, wings were transferred in a droplet of deionized water onto a non-adhesive substrate, typically the protective liner of double-sided tape. Excess water was removed, and wings were arranged flat without overlap. A strip of Tesa ECO & ULTRA tape (66 m × 50 mm)—selected for minimal autofluorescence, as it remains attached throughout subsequent staining, mounting, and imaging steps—was gently pressed onto the wings and lifted, ensuring wing adhesion to the tape. The taped wings were overlaid with a second tape strip and pressed gently to preserve structural integrity. The two tape layers were then carefully separated, thereby splitting the dorsal and ventral wing epithelial layers. Finally, the separated wing layers, still tape-mounted, were post-fixed for 20 min at RT in 4% formaldehyde in PBTT.

Wings were then briefly rinsed twice by rapid addition and removal of 500 µL freshly prepared PBS with 0.1% Tween-20 (PTw), followed by two 10 min washes in the same buffer. Wings were incubated for 1 h at 37 °C with RNase A diluted at 1:100 in PTw, then transferred to a 0.05 M HCl solution for 5 min to denature DNA and enhance probe penetration.

Wings were washed sequentially for 15 min each in 4:1, 1:1, 1:4, and 0:1 mixtures of PTw and FISH wash buffer (Fwb; 35% formamide, 0.1% Tween-20, 4X SSC). Fwb was prepared fresh before use and stored at 4 °C for a maximum of 48 h.

Wings were incubated with probes diluted to a final concentration of 3 ng/µL each in hybridization buffer (50% formamide, 5X SSC, 0.1% Tween-20, 100 µg/mL denatured herring sperm DNA, 50 µg/mL heparin). The samples were heated at 87 °C for 20 min in a thermocycler, then cooled gradually to 37 °C over 2 h. Hybridization was performed overnight for 12–16 h at 37 °C.

Following hybridization, wings were rinsed briefly, then washed twice for 1 h each at 37 °C in pre-warmed Fwb. Nuclei were stained with 20 mM Hoechst diluted 1:500 in PTw for 5 min, followed by four 10 min washes in PTw at RT. Wings were mounted in Vectashield, flattened under a coverslip using gentle pressure applied with magnets, and sealed with nail polish.

### 2.8. Nanorulers Calibration Standards

DNA origami nanorulers (GATTAquant GmbH, Munich, Germany) were employed as ground truth standards for validation of distance measurements [21]. These nanostructures consist of self-assembled DNA origami scaffolds with precisely positioned fluorescent dye attachment sites labeled with 40 ATTO 565 and 40 ATTO 633. Nanorulers with defined inter-dye separations of 120 nm and 160 nm were used to assess distance measurement accuracy, while a design with intermingled 40 ATTO 565 and 40 ATTO 633 dyes, effectively representing zero separation, served as a colocalization standard to evaluate measurement precision. Mounted slides were provided directly by the manufacturer. For each nanoruler type, images were acquired under conditions identical to those used for *Drosophila* wing samples.

### 2.9. Confocal Imaging

Hoechst, ATTO 565, and ATTO 633 channels were acquired on a Zeiss LSM 780 confocal microscope (Carl Zeiss Microscopy GmbH, Jena, Germany) using a Plan-Apochromat 40× oil immersion objective (NA 1.4). To minimize inter-channel displacement, line switching mode was employed instead of frame switching. Images were acquired with laser power of 0.2% (405 nm), 15% (565 nm), and 27% (633 nm); dwell time of 1.57 µs; voxel size of 103.8 nm × 103.8 nm × 250 nm; 16-bit depth. The pinhole was set to 1 Airy unit (AU) for the 488 nm wavelength.

### 2.10. Deep Learning-Based Detection of Nuclei and Spots

Nuclei and Oligopaint spots were detected using two independently trained deep learning models [19] applied to individual 2D planes of 3D image stacks, producing volumetric segmentations. Models were trained using EPySeg [22], with data augmentation (rotations, flips, intensity scaling). Probabilistic output maps were thresholded to yield binary masks for nuclei and point coordinates for spots.

### 2.11. Subpixel 3D Spot Centroid Extraction via Gaussian Fitting

Subpixel 3D coordinates of detected spots were refined using the Big-FISH Python library [23], providing initial coordinates and estimated spot sizes for a 3D Gaussian fit. This procedure yielded precise centroids even in the presence of moderate background noise.

### 2.12. Spot Pairing Across Channels

Before applying chromatic aberration correction, corresponding Oligopaint spots were paired across channels based on spatial proximity. For each image, detected 3D spot coordinates were used to identify the nearest neighbor in the other channel. When applicable, only spots located within segmented nuclei were considered for pairing, thereby excluding non-genomic or non-specific signals. The resulting paired centroids serve as the input for chromatic aberration corrections.

### 2.13. Linear Chromatic Correction (LCC)

To correct for chromatic aberrations and ensure unbiased distance measurements, we applied a method we named the Linear Chromatic Correction [14,24,25]. The principle underlying this correction is that, for paired FISH Oligopaint centroids, the mean difference between corresponding points should be zero in the absence of chromatic aberration—the pairs should have no preferred orientation—regardless of whether the points are colocalized or distant [14,24].

The correction procedure begins by computing the difference between each pair of centroids. The mean of these differences is subtracted to center the distribution at zero. For each spatial axis, a linear regression model is fitted to estimate the scaling bias. One set of points is then corrected using these per-axis scaling factors. Finally, any residual global translation is removed to ensure that the corrected differences remain centered at zero.

### 2.14. Affine Chromatic Correction (ACC)

In addition to LCC, we implemented an independent global method to correct chromatic aberrations by aligning spot coordinates across channels using a 3D affine transformation. This approach compensates for systematic inter-channel discrepancies, including global translations, anisotropic scaling, rotations, and shear.

The procedure operates on paired, colocalized Oligopaint centroids extracted from multiple images sharing the same chromatic aberration profile, acquired on the same microscope within a limited time window. To reduce the influence of mismatched pairs, pairwise distances are filtered using the median and interquartile range (IQR), removing extreme outliers that could strongly bias the affine transformation. Filtered paired centroids from all images are then aggregated to estimate a single global affine transformation matrix.

The affine transformation is estimated by minimizing the squared displacement between corresponding points, allowing for translation, rotation, anisotropic scaling, and shear. The transformation matrix is computed using an implementation adapted from a library by C. Gohlke [26], which solves the resulting least-squares point-set alignment problem using a singular value decomposition (SVD)-based approach. Once estimated, the transformation is applied to all points in one channel to generate corrected coordinates.

The resulting affine transform is stored and can be reapplied to images of non-colocalized spots acquired under the same conditions and within short time intervals, allowing consistent correction across datasets.

### 2.15. Distance Measurements and Resolution Assessment

Euclidean distances between registered, colocalized spots were calculated in 3D to assess spatial resolution. Distances for non-colocalized spots were similarly computed to quantify inter-locus spacing. Distributions were visualized using histograms and violin plots in Matplotlib (version 3.10.8) and Seaborn (version 0.13.2) [27,28], and robust statistics (median and interquartile range, IQR) were used to limit the influence of outliers and assess chromatic correction precision and spatial organization.

## 3. Results

### 3.1. Quantifying and Correcting Chromatic Aberration to Establish the Baseline Resolution

**Figure 1:**
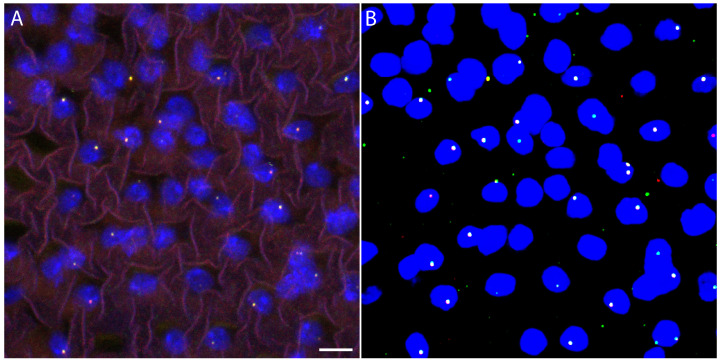
Colocalization control dataset for quantifying chromatic aberration in multi-color Oligopaint FISH. (**A**) Maximum-intensity projection of a cropped nuclear region from a representative 3D confocal Oligopaint FISH image, in which the same genomic locus is labeled with two fluorophores (ATTO 565, red; ATTO 633, green), serving as a colocalization control. Nuclear DNA is stained with Hoechst (blue). Under ideal conditions, the two FISH signals should spatially overlap. Scale bar, 5 µm. (**B**) Corresponding maximum-intensity projection of the deep learning-based segmentation output for the same cropped region, showing automated detection of the nucleus and individual Oligopaint FISH spots while preserving channel color coding. This comparison illustrates the ability of the deep learning models to identify nuclear boundaries and colocalized FISH signals in multi-channel 3D microscopy data.

To establish the baseline resolution of our imaging system and quantify the impact of chromatic aberration on distance measurements, we performed colocalization experiments in which the same ∼10 kb endogenous genomic region adjacent to the attP18 landing site was labeled with two distinct fluorophores using Oligopaint probes (Figure 1; see Section 2). Because both probe sets target the identical locus, any observed separation reflects technical artifacts rather than true biological distances.

**Figure 2:**
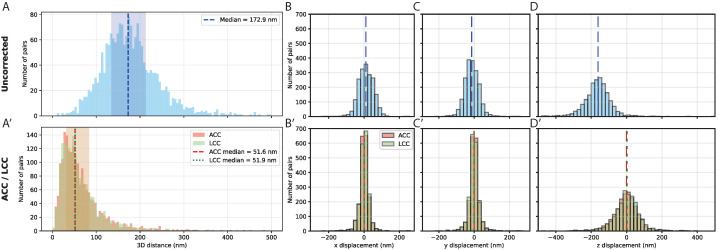
Chromatic aberration in multi-color FISH and its correction. (**A**) Histogram of 3D inter-channel distances between dual-labeled Oligopaint probes targeting the same genomic locus. Uncorrected measurements show substantial systematic offsets, with a median separation of 172.9 nm (IQR: 135.2–212.3 nm). (**A′**) Same distances after chromatic aberration correction. Affine (ACC, salmon) and linear chromatic aberration correction (LCC, green) both reduce the median 3D separation to ∼52 nm, with corresponding interquartile ranges shown as shaded areas. Medians are indicated by dashed (ACC) and dotted (LCC) lines. (**B**–**D**) Component-wise displacement histograms for uncorrected data along the x (**B**), y (**C**), and z (**D**) axes. Lateral displacements are small (median ∼30 nm), whereas axial displacement is larger (median 162.5 nm). (**B′**–**D′**) Component-wise displacements after correction using ACC (salmon) and LCC (green). Both methods reduce systematic offsets, with residual median displacements of ∼13 nm in x and y and ∼40 nm in z, reflecting the practical resolution limit of the system. Histograms are normalized to the number of spot pairs; shaded regions indicate the interquartile range. Data are shown for n=1917 spot pairs derived from 8 images of a single wing (one animal).

Analysis of 1917 dual-labeled spots revealed substantial systematic offsets between channels before chromatic aberration correction (Figure 2A). The distribution of measured distances showed a median value of 172.9 nm (IQR = 135.2–212.3 nm), with some spot pairs exhibiting apparent separations exceeding 500 nm. Chromatic aberration was highly anisotropic: lateral (XY) components showed median offsets of 29.7 nm in x and 27.3 nm in y (Figure 2B,C), whereas the axial (Z) component exhibited a median offset of 162.5 nm (Figure 2D). This reflects wavelength-dependent refractive index effects and the lower axial resolution of confocal microscopy.

Application of chromatic aberration correction substantially reduced inter-channel distances. Both the affine (ACC) and linear (LCC) correction methods decreased the median 3D distance from 172.9 nm (uncorrected) to 51.6 nm (IQR: 32.2–82.9 nm) for ACC and 51.9 nm (IQR: 32.2–82.7 nm) for LCC, indicating a marked reduction in systematic offsets (Compare Figure 2A and Figure 2A′). Component-wise median displacements after correction were similar between methods: 12.8–13.1 nm in x, 13.6–13.7 nm in y, and 39.7–40.5 nm in z (Figure 2B′–D′). This close agreement confirms both methods accurately model chromatic aberration. Residual distances likely reflect localization uncertainty, probe accessibility differences, and stochastic variation in probe positioning.

Analysis of residual chromatic displacements after affine correction revealed no obvious position-dependent variation (Figure S1A,B), indicating that the global correction captures the majority of systematic offsets. Decomposition of the 3D displacement vectors confirmed strong anisotropy, with lateral (XY) components showing no preferential in-plane orientation bias, whereas the axial (Z) component dominated (Figure S1C). The consistent agreement between correction methods throughout subsequent experiments further provides internal validation of measurement accuracy.

### 3.2. Validation of 3D Distance Measurements Using Genomic Standards and DNA Origami Nanorulers

**Figure 3:**
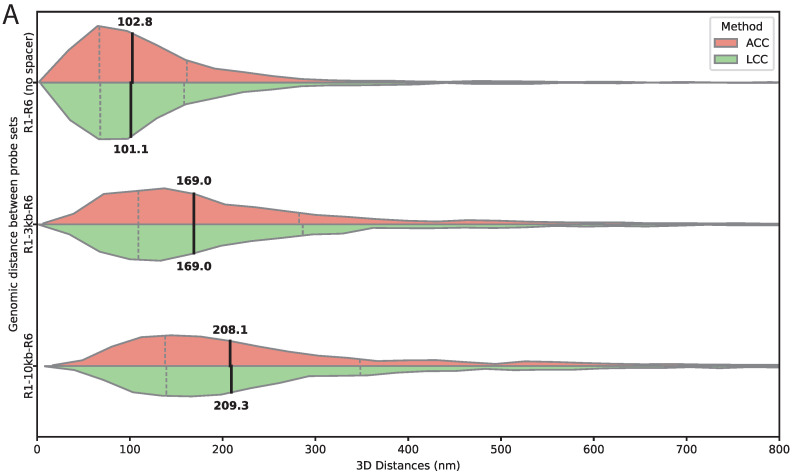

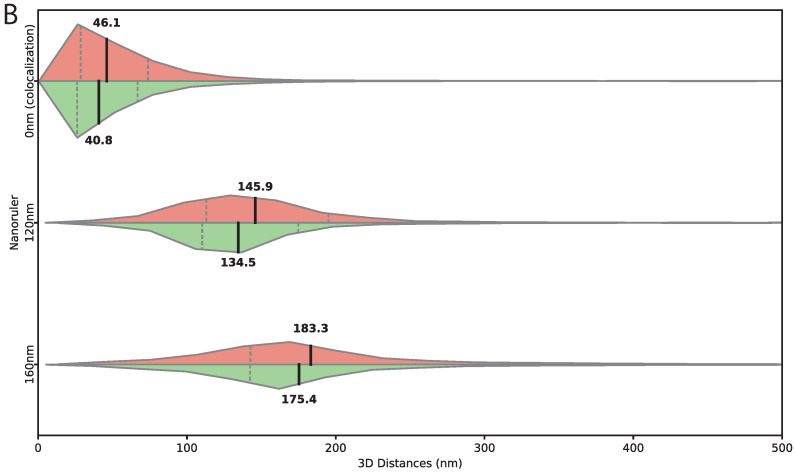
Validation of 3D distance measurements using genomic spacers and DNA origami nanorulers. (**A**) Measured 3D distances between two Oligopaint probe sets targeting R1 and R6 sequences flanking defined spacers of 0 kb, 3 kb, and 10 kb. Distances were measured after chromatic aberration correction using either the affine (ACC, salmon) or the linear chromatic aberration correction (LCC, green). Horizontal split violin plots show the distribution of distances for each spacer length and correction method, with medians and interquartile ranges indicated by internal lines. Distances increase systematically with spacer length, and the close agreement between methods demonstrates the accuracy of both chromatic aberration corrections. Numbers of analyzed spot pairs were n=2667 (0 kb), n=2363 (3 kb), and n=2606 (10 kb). These spot pairs were derived from 2 wings (2 animals) and 19 images for the 0 kb spacer, 2 wings (2 animals) and 18 images for the 3 kb spacer, and 4 wings (4 animals) and 29 images for the 10 kb spacer. Throughout this and subsequent figures, x axes are truncated for visualization purposes; measurements outside the displayed range are included in the analysis. (**B**) 3D distances measured for GATTAquant DNA origami nanorulers with defined fluorophore separations of intermixed 0 nm (colocalization control), 120 nm, and 160 nm. Horizontal split violin plots show distance distributions after chromatic aberration correction using ACC (red) and LCC (green). Both methods accurately reproduce the expected distances, confirming that chromatic aberration correction reliably captures absolute spatial separations. Numbers of analyzed spot pairs were n=17,239 (0 nm), n=6460 (120 nm), and n=4290 (160 nm). These spot pairs were derived from 1 slide and 2 images for the 0 nm nanoruler, 1 slide and 1 image for the 120 nm nanoruler, and 1 slide and 1 image for the 160 nm nanoruler.

To evaluate the accuracy of 3D distance measurements under biological conditions, we used a transgene containing two Oligopaint target sites, R1 and R6, labeled with ATTO 565 and ATTO 633, separated by 0 kb, 3 kb, or 10 kb spacers. Chromatic aberrations in these images were corrected using an affine transformation derived from a parallel colocalization experiment acquired under identical imaging conditions. After correction, the measured 3D distances between the two labeled regions increased systematically with spacer length (Figure 3A). For the 0 kb spacer, median distances were 102.8 nm and 101.1 nm for the ACC and LCC methods, respectively. For the 3 kb spacer, median distances increased to 169.0 nm with both methods, and for the 10 kb spacer, they reached 208.1 nm and 209.3 nm.

While the dual-targeted transgene provides qualitative insight into chromatic aberration and distance measurement precision, definitive ground-truth validation of absolute distances was achieved using GATTAquant DNA origami nanorulers [21]. Three types of nanorulers were employed, including one with intermixed ATTO 565 and ATTO 633 fluorophores suitable for registration and resolution assessment, and two with fixed inter-fluorophore separations of 120 nm and 160 nm. For each nanoruler type, we imaged over 4000 individual structures and measured 3D distances after chromatic aberration correction using both ACC and LCC methods. Due to technical constraints, calibration could not be performed using the intermixed nanoruler; instead, registration was derived from dual-color colocalization experiments on wing samples.

For the intermixed nanoruler, the median measured 3D distance was 40.8 nm (IQR: 26.2–66.8 nm) using LCC and 46.1 nm (IQR: 28.6–73.8 nm) using ACC (Figure 3B). For the 120 nm nanoruler, LCC yielded a median distance of 134.5 nm (IQR: 110.3–174.8 nm), while ACC measured 145.9 nm (IQR: 113.1–195.2 nm). For the 160 nm nanoruler, LCC produced a median distance of 175.4 nm (IQR: 142.5–304.8 nm), with ACC yielding 183.3 nm (IQR: 142.7–335.1 nm) (Figure 3B). Both correction approaches accurately reproduced expected distances—and distance differences—across nanoruler sizes, confirming the reliability of the measurements.

### 3.3. Target Sequence Length and Composition Affect Spatial Resolution

**Figure 4:**
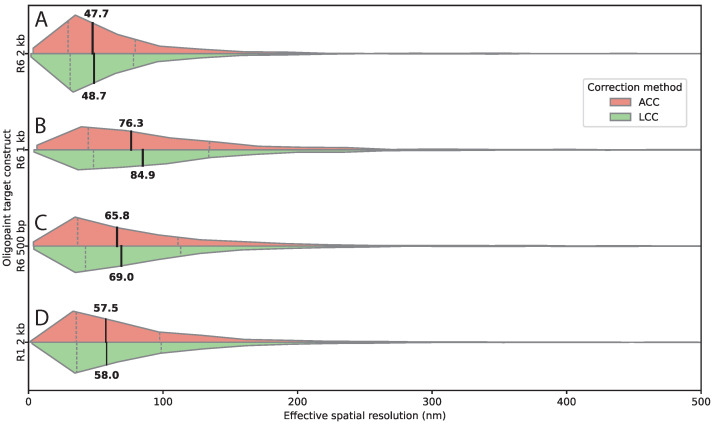
Target sequence length and composition determine effective spatial resolution in FISH. (**A**–**D**) Horizontal split violin plots of 3D inter-channel distances measured for Oligopaint probes targeting the same genomic locus, after affine (ACC, salmon) and linear chromatic aberration correction (LCC, green). (**A**) R6 2 kb; (**B**) R6 1 kb; (**C**) R6 500 bp; (**D**) R1 2 kb at the same locus but with a different sequence. Shortening the target sequence increases the measured inter-channel distance, indicating reduced effective spatial resolution. The alternative 2 kb sequence (R1) yields slightly larger distances compared to R6, highlighting sequence-dependent effects on localization precision. Medians and interquartile ranges are indicated on the plot. Numbers of analyzed spot pairs were: R1 2 kb, n=5409; R6 2 kb, n=1042; R6 1 kb, n=803; R6 500 bp, n=641. These spot pairs were derived from 4 wings (4 animals) and 42 images for the R1 2 kb construct, 2 wings (2 animals) and 14 images for the R6 2 kb construct, 2 wings (2 animals) and 14 images for the R6 1 kb construct, and 5 wings (5 animals) and 38 images for the R6 500 bp construct.

To assess how target sequence length—at constant probe density—affects resolution, we used transgenes containing the full 2 kb R6 region and truncated versions of 1 kb and 500 bp, co-labeled with ATTO 565 and ATTO 633. For the 2 kb construct, median residual distances were 47.7–48.7 nm (Figure 4A). The 1 kb construct showed substantially increased distances of 76.3–84.9 nm (Figure 4B), while the 500 bp construct yielded 65.8–69.0 nm (Figure 4C).

Target length strongly affected detection yield. Average detected spots per image decreased from 190.5 spots (2kb) to 57.4 (1 kb) and 34.0 (500 bp) (Table 1), reflecting reduced probe binding sites and signal intensity.

To assess whether factors other than target length contribute to measurement performance, we analyzed an additional 2 kb construct, R1, inserted at the same genomic locus but exhibiting a different DNA sequence compared to R6. Importantly, this construct was targeted by the same number of Oligopaint probes as the original 2 kb construct, ensuring comparable probe density and labeling strategy. Despite these similarities, the alternative sequence yielded fewer detected pairs per image (128.8 versus 190.5, Table 1) and slightly larger residual inter-channel distances (compare Figure 4A and Figure 4D). This indicates that sequence-dependent effects—potentially related to hybridization efficiency or local chromatin organization—can modulate measurement performance even when target length and probe number are constant.

### 3.4. Probe Density Influences Detection Efficiency and Spatial Resolution

To evaluate the impact of probe density on measurement performance, we systematically varied the fraction of fluorescently labeled Oligopaint probes targeting a fixed 2 kb genomic region by adjusting the ratio of labeled to unlabeled probes while keeping the total probe mass constant. This approach preserves hybridization conditions while progressively reducing the number of fluorescent probes contributing to the detected signal.

Three labeling conditions were examined, corresponding to approximately 100%, 66%, and 50% fluorescent labeling. Under these conditions, the 2 kb R6 locus could be covered by up to 87, 57, or 43 fluorescently labeled Oligopaint probes, with the remainder of the locus filled by 0, 30, or 43 unlabeled Oligopaint probes. Fully labeled conditions yielded an average of 190.5 spots per image (Table 2). Reducing the labeled fraction to ∼66% decreased detection to 133.2 spots per image, and further reducing it to ∼50% resulted in only 51.1 spots per image (Table 2). Reducing the fraction of labeled probes lowers fluorescence signal, diminishing both detection efficiency and localization precision, highlighting the strong dependence of measurement performance on the number of fluorescent probes present.

**Figure 5:**
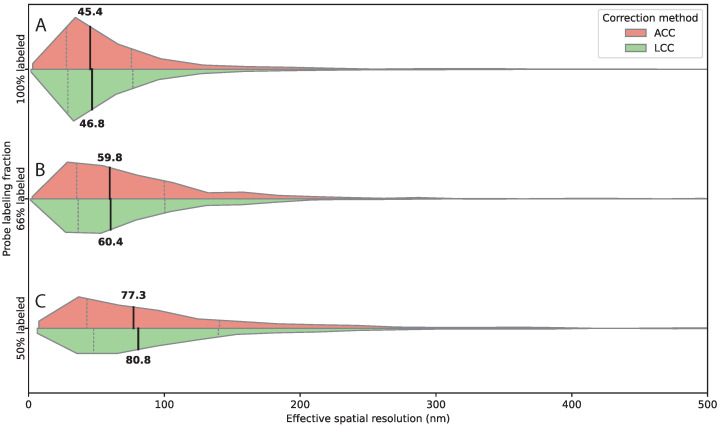
Probe density modulates detection efficiency and effective spatial resolution. (**A**–**C**) Horizontal split violin plots showing distributions of 3D inter-channel distances measured after affine (ACC, salmon) and linear chromatic aberration correction (LCC, green) for decreasing fractions of labeled probes targeting the same 2 kb genomic region. (**A**) 100% labeled probes, (**B**) ∼66% labeled probes, (**C**) ∼50% labeled probes. Medians and interquartile ranges are indicated by the internal quartile lines. Reducing the fraction of fluorescently labeled probes lowers effective spatial resolution, as shown by the systematic increase in median distances with decreasing labeling fraction. This trend is consistent across both correction methods, demonstrating the robustness of the measurements. Numbers of analyzed spot pairs were: 100% labeled, n=2667 (2 wings, 2 animals, 14 images); 66% labeled, n=2131 (2 wings, 2 animals, 16 images); 50% labeled, n=971 (2 wings, 2 animals, 19 images).

Spatial resolution also worsened with decreasing labeling fraction, although less sharply than detection efficiency. After affine chromatic aberration correction, the median residual distance increased from 45.4 nm at 100% labeling to 59.8 nm at ∼66% labeling and 77.3 nm at ∼50% labeling (Figure 5). Comparable values were obtained using linear chromatic aberration correction, with median residual distances of 46.8 nm, 60.4 nm, and 80.8 nm, respectively (Figure 5), demonstrating that this trend is robust across correction methods.

## 4. Discussion

### 4.1. FISH-Dist Enables Reproducible 3D Measurements of Short-Range Genomic Distances

We developed FISH-Dist, an automated pipeline designed for quantitative 3D distance measurements in FISH imaging at the kilobase scale. FISH-Dist combines deep learning-based spot detection, sub-pixel 3D localization, and dual chromatic aberration correction methods (affine and linear), with performance assessed using calibrated standards such as DNA origami nanorulers.

While many existing FISH tools focus on large-scale chromosomal structures (hundreds of nanometers to micrometers), biologically critical interactions—such as enhancer–promoter contacts, local chromatin loops, regulatory clustering within topologically associating domains, DNA damage foci, and homologous chromosome pairing—occur at much shorter distances (10–50 kb). At these scales, chromatic aberration and localization precision are limiting factors. In our system, uncorrected inter-channel offsets exceeded 170 nm, with axial components approaching 160 nm—comparable to or larger than many true biological separations. FISH-Dist reduces these errors to approximately 50 nm for colocalized targets.

Additionally, we demonstrate that 2 kb probe targets offer an optimal balance between detection efficiency and spatial compactness, achieving ∼45 nm resolution. These advances enable robust, quantitative measurement of short-range genomic interactions that are typically inaccessible to conventional FISH analysis pipelines.

### 4.2. Probe Design Guidelines Emerge from Systematic Parameter Testing

Our systematic evaluation of probe design parameters yielded several actionable insights for experimental optimization. The relationship between target sequence length and resolution reveals a critical trade-off: longer sequences improve detection efficiency but compromise localization precision. Our 2 kb construct achieved optimal 45 nm resolution with robust detection (190.5 spots/image). Shortening to 1 kb degraded resolution to 75–80 nm despite reduced target size. The 500 bp construct showed slightly better resolution (∼70 nm) than 1 kb but both exhibited markedly reduced detection efficiency.

These findings indicate that competing effects exist, with shorter targets providing compact labeling but reduced signal intensity and fewer probe binding sites, both of which compromise localization. The 2 kb length represents a favorable compromise. Importantly, sequence composition influences performance even at fixed length, as the R1 construct showed reduced efficiency despite having the same length and number of probes as R6. Factors such as GC content, secondary structure, or chromatin accessibility likely modulate hybridization efficiency.

Probe density experiments showed reducing labeling from 100% to 66% caused moderate degradation (45 nm to 60 nm resolution, 30% detection loss). Further reduction to 50% caused substantial degradation (80 nm resolution, 75% detection loss). Even with probe excess, reducing fluorescent fraction diminishes photon counts, degrading centroid localization. For high spatial accuracy, we recommend maintaining at least 57 oligos/fluorphores per probe set spanning 2 kb (corresponding to ≥66% labeling in our conditions).

Our optimization was conducted in specific genomic and cellular contexts. Absolute values likely depend on chromatin accessibility, nuclear architecture, and cell type. However, general principles—optimal target length balancing signal and compactness, sequence-dependent performance, sensitivity to probe density—should apply broadly. We encourage pilot optimization in specific biological systems.

### 4.3. Methodological Considerations and Limitations

Validation with DNA origami nanorulers provides ground-truth accuracy under idealized conditions, while transgene-based measurements in fixed biological samples demonstrate accuracy in realistic contexts where chromatin organization and nuclear architecture are preserved. This combination represents a strength of our approach, as it bridges artificial calibration standards and biologically relevant measurements. Nevertheless, probe accessibility can vary with chromatin state, DNA sequence, and cell cycle stage. Although fixation stabilizes chromatin structure, labeled DNA retains conformational heterogeneity reflecting the ensemble of chromatin configurations present at fixation, such that measured distances represent spatial averages rather than rigid, single conformations.

Our chromatic aberration correction assumes a stable optical transformation between channels during image acquisition, and performance may degrade in samples with substantial axial drift or stage movement. In addition, the deep learning-based spot detection relies on training data representative of the target application. Although the trained model generalizes well across the range of probes, labeling densities, and imaging conditions tested here, users working with substantially different probe types or imaging modalities may benefit from retraining or fine-tuning the model. Due to their size, the training datasets and scripts are not publicly distributed but are available upon request.

Throughout validation and optimization, we employed two conceptually distinct chromatic aberration correction approaches, affine and linear, which consistently produced nearly identical corrected distances across all tested conditions, typically differing by only a few nanometers. This close agreement indicates that both methods accurately model the chromatic aberration in our imaging system and provides an internal consistency check that strengthens confidence in measurements.

Although the two approaches differ primarily in workflow rather than performance, maintaining both in the analysis pipeline offers important diagnostic value. Agreement between methods, as observed here, confirms optical stability and the adequacy of global correction models, whereas substantial divergence would indicate optical instability or model inadequacy and prompt re-evaluation of imaging conditions. Consistent with this interpretation, spatial analysis of residual displacements revealed relatively uniform chromatic aberration fields across the imaging volume, indicating that global correction models capture most systematic offsets in our system. Users are nevertheless encouraged to assess spatial uniformity in their own setups to determine the most appropriate correction strategy.

### 4.4. Comparison with Existing Methods

Several computational tools exist for FISH image analysis, each with distinct design priorities and validation strategies. Classical approaches based on watershed segmentation or intensity thresholding remain widely used, but they often require manual parameter tuning or segmentation, which is error-prone and can lead to substantial over-segmentation, particularly under variable background conditions or in regions with densely packed spots.

Particle tracking frameworks such as TrackMate [29] provide flexible and sophisticated spot detection and localization, capable of handling multiple spots per frame. Tools like the DNA-FISH Fiji plugin [30], which leverages TrackMate for spot detection, offer semi-automated analysis of FISH images, including distance measurements between genomic loci. However, in noisy images such as ours, these methods can generate excessive false-positive detections. While post-processing can mitigate this, the procedure is often slow and not optimized for high-throughput FISH distance measurements. Additionally, neither TrackMate nor the DNA-FISH plugin explicitly addresses chromatic aberration correction, which is critical for accurate short-range distance quantification.

FISH-quant [23] is the most directly comparable tool. However, it was developed primarily for RNA FISH and transcript quantification rather than precise genomic distance measurements and does not include chromatic aberration correction. In contrast, FISH-Dist is specifically designed for short-range genomic distances, implements two complementary chromatic aberration correction methods (linear and affine), and has been rigorously validated using both transgenic constructs and DNA origami nanorulers. Its systematic evaluation of probe design parameters provides increased confidence in quantitative accuracy compared with single-method or less extensively validated pipelines.

## 5. Conclusions

FISH-Dist has been developed with accessibility and reproducibility in mind. The complete analysis pipeline is available as open-source software, accompanied by comprehensive documentation, example datasets, and tutorials to facilitate adoption. The tool is provided as a command-line application and includes pre-trained deep learning models that support many standard use cases, while also allowing retraining or fine-tuning for specialized experimental conditions.

More broadly, FISH-Dist provides a validated and automated framework for quantitative 3D distance measurements in genome organization studies. By integrating chromatic aberration correction, robust spot detection, and systematic distance analysis into a single workflow, the pipeline reduces technical barriers and enables reproducible, high-throughput spatial measurements across diverse experimental systems.

## Figures and Tables

**Table 1:** Average number of detected pairs per image for different genomic constructs.

Construct	Target Size	Detected Pairs per Image
R6	2 kb	190.5
R6	1 kb	57.4
R6	500 bp	34
R1	2 kb	128.8

**Table 2:** Detection efficiency as a function of probe labeling fraction.

Labeled Probe Fraction	Fluorescent/Unlabeled Oligonucleotides per Locus	Detected Pairs per Image
100%	87/0	190.5
66%	57/30	133.2
50%	43/43	51.1

## Data Availability

The FISH-Dist pipeline is available as open-source software under the BSD license at https://github.com/baigouy/FISH-Dist (accessed on 27 January 2026). Pre-trained TensorFlow deep learning models are automatically downloaded upon first use. Comprehensive tutorials and documentation are available at https://fish-dist.readthedocs.io/en/latest/ (accessed on 27 January 2026). Due to their large size, example and training datasets are not publicly distributed but can be made available upon request. The sequences of all constructs and oligos used in this study are provided in Supplementary File S1 (Supplementary_Sequences.pdf).

## Supplementary Materials

The following supporting information can be downloaded at: https://www.mdpi.com/article/10.3390/bioengineering13030268/s1, Figure S1: Spatial distribution of residual chromatic displacements after correction; Supplementary File S1: Sequences of constructs and oligonucleotides used in this study.
