# Supplementary material for: FISH-Dist: An Automated Pipeline for 3D Genomic Spatial Distance Quantification in FISH Imaging
Source: Bioengineering (Basel). 2026 Feb 26;13(3):268. doi: 10.3390/bioengineering13030268 (PMC13023453; doi:10.3390/bioengineering13030268)
Supplement: Supplementary file 1 [file bioengineering-13-00268-s001.zip › Figure S1.pdf]

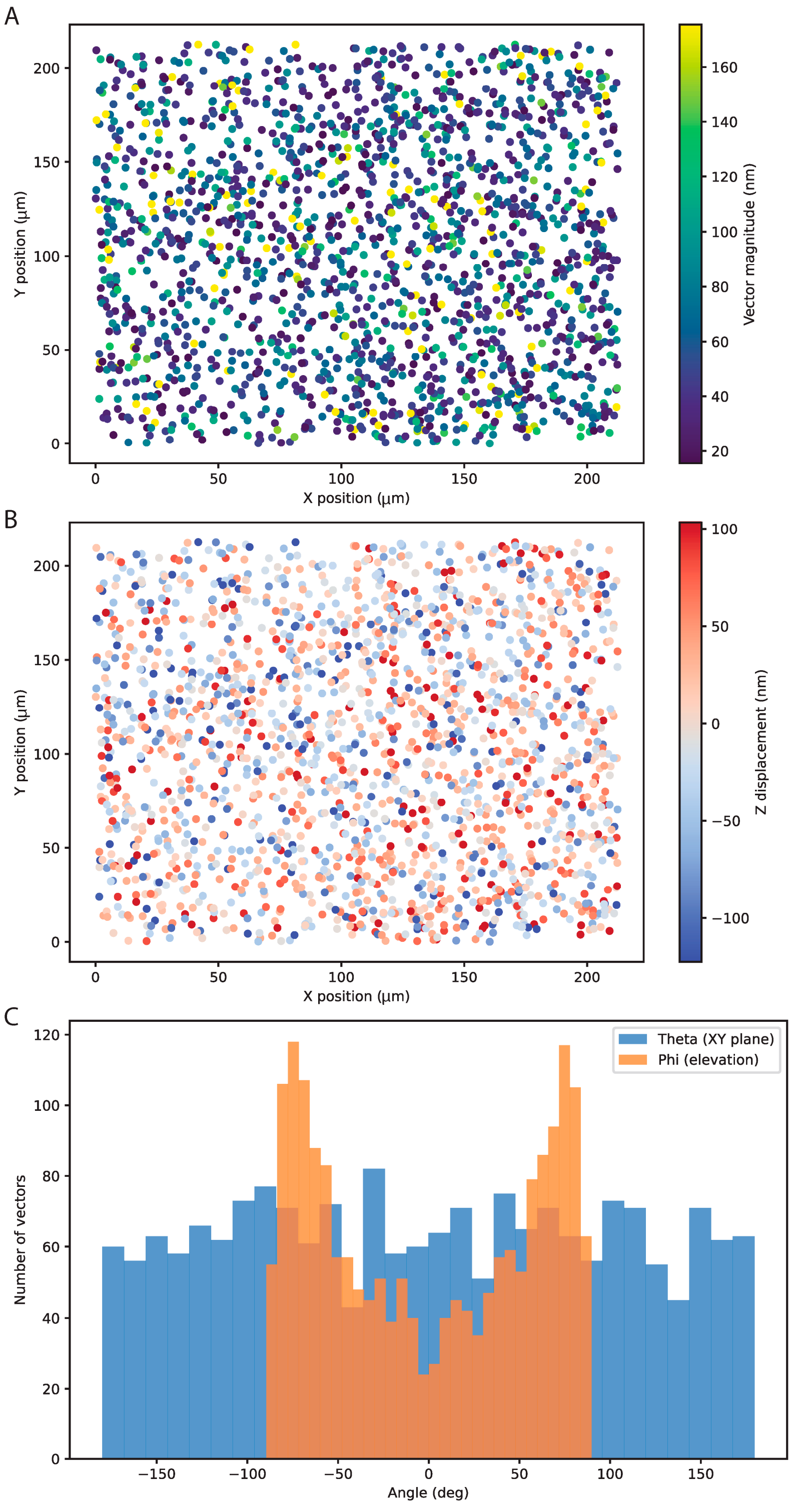

Figure S1. Spatial distribution of residual chromatic displacements after correction. (**A**) Midpoint positions of dual-labeled loci across the XY field, colored by the magnitude of the 3D displacement vector after chromatic aberration correction. Displacements were clipped at the 5th and 95th percentiles for visualization. (**B**) Same midpoint positions colored by the Z-component of the displacement, highlighting the predominance of axial offsets. Color scale clipped at the 5th and 95th percentiles. (**C**) Histogram of vector directions. Azimuthal angles in the XY plane (theta) show nearly isotropic lateral displacements, whereas elevation angles relative to the XY plane (phi) reveal the strong contribution of the axial (Z) component. These panels indicate that residual chromatic aberration is fairly uniform across the imaging field, supporting the use of a global correction. Data are shown for (n = 1917) spot pairs derived from 8 images of a single wing (one animal).
