# Supplementary material for: FISH-Dist: An Automated Pipeline for 3D Genomic Spatial Distance Quantification in FISH Imaging
Source: Bioengineering (Basel). 2026 Feb 26;13(3):268. doi: 10.3390/bioengineering13030268 (PMC13023453; doi:10.3390/bioengineering13030268)
Supplement: Supplementary file 1 [file bioengineering-13-00268-s001.zip › Supplementary_Sequences.pdf]

>R1
GTCAGACGCAGAATAATAATAATTAAGCAATACGTGTCACCCGAATGACGACCCCTATACATCGCTCT
CCAGAGTATAATATTAAAGGCTTCATGGCGTTATAGTAGTTGACTGGTTACTGAGACTATCGCATATA
GGTAGGTAAAGCGTCATTGTAATAGTACGGTTTAGCTTCATCACTTTTTATCAATCTCAAGTTTCTCGC
CGTTTAGTAGTTCGACACGTTAATTAAGCGTATAACGGCATCAGTCTACACGCTACCCGTATTCGGGA
CCGCGCGAAGAATTATTACTCTTTTATATATTCAAGGAAGTGAGTGATGTAAAATATATTTACGTAGG
TTCTCGACCGCGGCCCATCGTTACCACATAAAGGTATATTAAATAGACGACCCCGCGCCCGTAGGGA
TCATGCCTTATTATATAACTTAGCTGTAGGCTATTTTGACGTTAATCGGTCAGTACTGACTAGGTACTA
GCAATCTTCGACGTATATGGCTCTACGACGTCTTCAAAATTATATATTACGGTAGAGGGCCAAGCTCA
CATGAGATTATTATATTTTACTTCGCGCCCCTAGAAACAACAAATTATATGACGGTTAAAAGGGAATA
CTATGGCTCCAGCGTTGGAATGCCCGGCGGCTGTATCTATATATATTAAATTAACGGTCTAGAGCGA
ACCATTTCTTCCTATCTAAAACCGCGATACAAGTCTGGCCCTCTATTAATCTTTAACTCTTCCGGGTAA
GATTAGGATAACGGACGGTTTGAGTTTGTACCAGTAATCGGAGTGCGCTTAAATTTACGATGATGTT
ATAAAAAATCAGATCTCAACGGCTTTACTCACGATGCGATAGAGCTTGGGCCTCACATCCATATTATT
TATATTGTATAGAGGCAAACACTATTAATCTTAATTCAGATCTGGGGCGCCGGTAGTTGATCCTATGA
CCTTCAGCAACGGTTTACTGAAGTTCCTTATAACTCGGGCCGATTTCACGTGAATGTAATAATAATAA
TATAACGCCCTATGGGTATTACCCATAAGGGCGCGCTTGATTAACACATAACTTTACCACACCACAAA
ACGGAGAAGGTACAATATTATTATTAAGGGATGCCTATCCCTACACGAAAACTTTTAATATTTTAATC
CCCGGCATGAATCGATCTCAGGGGGCTTTAAAATTAGATATTACAGAGTCGGGGACTATTCCTTCTTA
TAAAATATAGTCGATATAAAGTGCCGTATAGGGGCTGGAAGATAATTATCTTTTACCTGCGCAGTTA
AGGATAACGGTTGGCAAGTCACATGAACTCGTCTGATCCAATTTATAAGAAAAAGTCACAACGAATA
TAATATAATTAAAATAAACGTCGGCCCCCCGACCATGAAGTTTCCATATTATAAGGGACGTAGCACCC
GGCTCAGTAATCCATTTGGCCTTGTGTAATATTACGATTCAGCGCACAGCAAATGACAGATGTAAGCT
AGCCCCTCGTGAATAACTGAAACTAGTTGTTCACCACAAATGGGCCTCAATAGGCTCATATAGTTCAG
TGATACATTCCAAATTAATTAATATAAATCAATGCTAGGCGCAGTGGCTTCCCCAGGACCTCCCCAGG
GTTGGCTCTTAACTCGGTCACTAAATATTTAATTCTTATATTTTCAGCGGAAGGTTGCGTGATATTTTA
ACACGAAGACTAGGACTGAGTCATTAATAAAACTATCAGTAAGAGACCGTACACAGCGTGTCAGGA
ATAGGACGCTACCAATAGTTGATAAAACATCGTAAGGGACTGTCCCCACCCAGCCCTGCAATATATAT
TAATCTTTAAACGAGCGATAATTTACCTATAAGAACTTATAACTTGTGCTATAGTCCCACAAGCCTATT
GATAGTAAGTGGAGTATCGAAGTATTAGATTCCTGTCGAGCCAATTTTAGAAGTACTTCCAACGTTCT
CTGTTCCTCGA

>R6
TACAATTTTATAGGGTTGTGTCTACTCATGGAGTAAGGTTAGCCATTACCGACTTCTCCAGAGATACC
CAACCTTTATTAGTATTATTAGCCGTGACCTTGACATATACACACCAACTAGTATAACAAATCGGCTG
GTTGAATTCTTTTATATTAATATCTAGTAGGGCTCGAGGTTAACCGAGACAAACATAAGATACAGCTA
TTATAAAAGAGTAGGGTGGCGAAACGATAGTCCAGATTTGTCCATAAACGTTATCATGTACTGGAAA
GGTTCCCATGGGAGACATAGAGTCAGGCCTTAATATTTAAAAAACGGTACTAACTCTAGTATAAAAG
ATTCAACGGAGACCACATTAGCTCCATGCCGAGCCCTTGATTACCGACATAATTTTTATAATCAAGCC
TGTCCCGGGCACAGCGCGTATAGTTCCTTATATTATTATTTCATATTCTATATATTATATTATGCCTAAC
CTAGCGTGTACTCGGAGAGATCTTGCTCTGTGAGAGGGCCTCTAACTCACATTAATTATAAATGTAGC
CCGATGTTCCTTAAATTAATTAGTATCCTGCGCCGAAGAGTGGATAACCAATCTTATAACAACTGTGG
TGTATGATTTGGAGCTAGAGCTACTGTCAATGTATATTTAACGTTAAAGGACCCCCATACGCTTTTCA
TCTGACCAAACAACGATCTTGGACTTAGTAATTGGCTCATGACAATTATAGATTGGATAATCCGACAC
TATTCCGACGCCTATCCGCGACAGGTAGGCGAGACCTAATGCCTATATTATTATTCGGTTAATTATCT
GGGATATTAAGGCGGATAATTATAAACCAGTCTGCAGCGTTTCAAAAGGACTAGTCGACGCTTTCAT

GTGTGAAGGATAATAAGTCAGGAAGCTTATTATTAATAATGATGTAGTGATGTAACGGCGGTTGTTT
TTGACAATAAGGTACTCAACATCCTACGTAAGAGTTCCTTGTCCAAGTGGCGTAATATTATTACGTCA
TAACAGCTAATCTAACACATGATGTGCAAAAGGGTTATGACTGTCCTAGAACCAATGCCCCAAAGTCT
GCGTTACTCTAGGACAATTTAACATTTTAGACGTGCCATATAATGCTTATATATTTAAGCTATGTATCG
AGAGCAGGGACCCCAGTTAAATATTTATATATGTTAGACCCTTCCGATGAAGTAGGTTGGACTATTTT
AATGATTTCATATGTAACGACCGCTGTAACTACGGGATGATCGGCTGCTAGCTCAGGTAATATATTTA
ACGATAACCTGTCTTTCCGTAATATAATTATTTGCAGTCGTCTATGATAACGGGATCGGCAGAAATGT
GGATTCTTACGTGATTCTGAAGTAATGTGTCGCGGAAAATATTATAATAATCCAGACCCGTTATTCTC
GTAGCTACGCCCTAAAGCGAATAATATGTTTTATACATTAGATGGCGTGACCGCCGAGTTGTCTGAC
GCACCTCCGTTAAAAATATATTGTAAATGAACCGTTCCTCTTAGCATGTAGAGTTTTCCTGATAATAAA
GTCTCAGATAGAGTATCCTCCGGATTAATATAATAATTTCAATCCATGGCCTTGGGTCAGCCCTTGGC
CAGTCTGTGGAAATAATATAGAGAACGTTTCTAGTGCCGGATTATCTTAAGGGGCGGTTAATTAAAT
ATTCTGATAGACATCGAGTTGGTCCCTTATTAGTGTACGGCTTTTGTCTCCTGATTAAACTGAGACTCG
TATATCTTTTATGAGTAGACCGCGTCGTTAGAAGAGTTAGTTATATTATTTATGCCCTACTGTCTAAAC
TGCCCGCTCCCTATTCGTAGTATAATTCTAGGTGCGCCCTCGGTTATAATTTTTGTAGCTTTAGTTATA
TTATATCATAGCCGACTTGGGAAAGGCTCAGCGGTTCACCAGATGGATGTTTAATATATAGAGAACC
GGTGTCAA

>3kb spacer (cloned in between R1 and R6) – random sequence from Xin Y, Le Poul Y et al PNAS 2020)
AAGTGGAGAGGATCCTGGTTCCTGTGGCCAGCATTCCGTAGGATGTATAATAACGGCGCACAGAGC
CTTCCAACCTCCCACTTCGTTATCATCTGACATGAGTCATGTTGCGGATCTTCAAGGTAACTCATACAC
ATCATTCCATAATGACTTTGATGACCTCATCGCTTTTAGTCGCCCACTTGGAATAATATTGATAGCAGT
GCATTATTGAGCCTTATCCGAGAGCCTCATAGGTGGCGTTTATATCTTGAGTACATATAAGTGCGCGG
CGTAGGGTTCACCATCTAAGTGCTGAAGCTAGGGGGTTCGCCGCGCCGGGCACGAGGATGGCAGTC
TCGGGGCCACGTGCATTAAGCTCGGTAACTCGGATGAAACTCGGTACCAGTGAGTCCACGTCAGGC
ACGGGAGTGCATATGGGGTATGAAGGTCTACAGAGACGCTCAATATGATTCTTCACCAATCAAGTTT
AGAGAAATAAAGATCGATATTTGTGGGGCACGGGTGTATACAGGATCTAGAGGACCGAATAAAATC
CGCTTTGTGCGTATGCCGATGCGTACCCGGCGAACAAACTACCTGAGAAAGCTTGTGGGACGCCTAA
GTGAGTACTAGCGGTCAAAAAGCCAGACGGGGTGACGCCGGGAAAAGGACCCGCCTGTTAGAGCA
CTTATCTTCTGTTCTGTGACATTTCAAGGGCTGGAATTCTCTGTTGGGACTCGGATCCTCTATGCCCGC
ATACGCCTCGTTGATGTATCCTGATTAAGCTAGGTCTCTTTGAACTTGTGCAGCTCCACGGATAGCC
GAACGTTTCGGAGTTTGTGTGTCTTTCTTCCATATGCTTCGTGTAATTACATTTATTCCACAAACAATA
AAATAGAGGGGACCTGTCTAAAGAACAACACATGGCAAAGTGGGAATACAACCAGAAAAGTGGTCC
AATAAACAAAGAACGTGAATCACTCAGGAATGAGAACCATCGTGAGCCTTCAGCAACAATTACCCAT
GGCATCTAAATGGCGAGTACTTTACAACGCCTGACAAAGATAGCTTACGAATCATGTGACGCGAGTA
TCAATAATTTTGTATGAGTCTCACCCAGATTCTGATCCGCCGTTAAGCTCACCCGTTAGGCAAACTCTT
TGGCATCGATGGTAGTTAGCTCCATGTAAACAATTCTTACTAGAGGTAGGCCCAGCGTGCGCGCGCT
TACCTATTGAGGGTTTGATCGCCCTTTAGTAGAGTCGGGGTCCGGCTTCAGGTATCGAATAGATGAT
CTGCTCTTGGATAGTGGCTGACAAAAGTACTAAGGGAAATCTTTATCCTTATACTAAGTCCGAGGAC
AGTGGGTATAGACATGGGAAGCACTCACTCAGATATGTATAGACACAGCAAATCGTGTATTTAAAAT
CAATTCCTTAGATTATTACGAAAAGATAAGAAACAGGGCAACACGACATTGGAACAGTTACGTAATT
GCGTTTTGCGGTTATGCAAAGTTTCATTTCGCTAGGAGTGTGGCCCATATGAGATTCATCCGCTTCTT
CTCGACGTGAGAGGGACCCCAGCTTTGCTTCTCTTAACAATAATCCTGTCGCTTAGGCAATTTTATGG
TGTCGTGTTTAATTGTTCAATACATCACATCCACGCTTATTCCTGTCGCTCCGCTCCATTTTATGCTCGA
ATCCAGCGTAGGCGGGTCATATGTGCCTCTTTATTGAGCAGTTGGGAGGTTCGTTTCTCGGGAGATG
TCGTTTTGACCATTGCCGATCCTCGGCGCCAGAGCGTGGAAATACCCCGACATCTGGCTTTCAACTTG

GCAAACCTCAGGGAGTCGTCATCACTTGACCTAGAACCCGGTCGGGGGCACCGATTATCTGCCTTAG
CCATTTCCGGTTATGGCGGAGCATATTATCAACGCGTGAAAATTCTGGCCACGTCTTACAAAGTGTCA
AATGGGAGCCCGAACGCCCACCGAGGCATACGATGCTGGCTAGCTCGTACCATTTATGAGATGAACT
AATCTGAACCTCACCTACTGGGTAATACGAATAAGTCACTATACTaAAATACTTTTCGGTAGCGAATA
AGCTATATATTACATAAATAAATGAGCTCAACAACCAGTGAGATGCACCAGGGGATTGGTTAAAAGC
TCGACCGACTGCTTTTTTAAATCGATGGAATATACTATCTTGAATCTTACATAAATTATTATGACAAAT
GACTTGAATTTAATGTAAAAATTATTTTACGTTAAAAAATTATAATGATGTCCAATGCAACCTAAGTC
GCGAAGACTCAGAATCAACGGCATCGGGGTGAAAGTTACAGTTTTACAGATACCGAGATCAATTTTC
CGTTTAATAAGTTGACTTCTGGTACACTAAACCAGTTAATGTTATAACAAAGTATGAAAGTTTGTAGA
CAAATAGCCTAGAACCGGACTGAGTACCTAATAATGTCAACGCGCGAGCAATCAAGATCTCGAAGAC
GAGGCGTACGCATTGCCCCGAGAGGCCATCTCAGGCGCGCACCCATGGTCCGAGAGCAACCCAGAT
TCAGAGTCAACCAAGAAGACGAGCCTAGCGATCAATTAAGTAAGGTAACTATAGATGAAACCAAAT
GGTATTGGAATGCAAAGCCAAACAACGGATGATCCTGACTGCACCCGAATAGAAGTTCGTATGGAC
ACAATGCGCTATCACCCGCCTGGCTGACGATCGCACGCCCCTAGCCCCCTCAAAGAGCAAGCTGCGG
TGATAACTGTACTGATGGCACTCTAAACCTGAACCCCGTTTCCGTCGTAACGAATGTATCAAAAACAC
GGCAACGTTCGGTTAGACGGACCGGACCA

>10kb spacer (cloned in between R1 and R6) – From Galouzis & Prud'homme, Science 2021)
ATCGATAATCGCCCGATTACCGCGCTGAGCGGTCTTAAAGACCCATAAGAAATGCGGCGATGGCGG
CTTTAGATAAGTAAGTCGTCGGGGCGCTCATAAATTTCGAGCGCGATCCACGGATTTATGCACTCGCT
GGAAAAGCTATTACCATTAGGCTTTTCGCGACCACGGATTTTTCCGCTTGCCTGAGACAAGTGCGGC
GCGGCAGTTGCAGGCAAATTATGTGTGAGGCAATGCCGCGGGCATGTCTACACCGAAATCAAATTA
CGGCAACCTCTATTCACTTATTTGCTTAGTTTTTTGCGCAGTGAGCGGCCAGCGCCTTGTATTGGCATC
TAATTATTCCGTTTAAGGACGCAATTTTCTGAGCTAAAACTCGCTTATGGAGAGATCTAAATTTCCCC
GCTTTTGGCTTGAATAAATTAATCGAATTCCCCGCTGGCTATTAAAACACACAAATGGCGCTCTCGTC
TGTTTCAATGTAAATTGCAAATTGCTCAATCCGCCTAATTGATGTGAGCCCATGCAATAGTTTTGTGCC
AATCATTTTTAGTACACCCCTAACTGGTGTTTTTTACGCATAATATGTGCCATGGCTTAGGGCCTTTTG
GTGGACTTACCAACTGAAGAAGACGATTGTGGGGGTGCGTTTGGCGCAGTGCACGCCTGCGAGCAG
GAAATCTCTTTCTCGGCCTGTCTGATTTTGGCCAAGACAAATAAATCCGGCTGGCAGATAGGCAGAG
GGGACCCGGCGGTCAGGGCCGTGGACATTGAACTTGAAAACGCAGCCAGCGCCGAAAACATTGTAT
TCAACGAACGGCAAGTGCTGCGCGGCATGGGTGTCTCTGGCTAAGGTTACGGCGGTTGGGCAACAG
GTTTTCCCCTGGCCAACACTGGGGGGAGAAAATAAAAAGGAAAATGTTCAGGCTGCCATAAGTGGG
GAAAAAGGAAAACAAAACATGAAACACGGGCCGGGCAATGTCACTCGGCATTCGCTTGATTTTCCGC
CTAACTCGCAGCGGTCCTGTGTGTAAATAATGTCTAATGTTGCATGCCGGTTGCATAATCGTGTGGCA
ATTATGCCAGAGAGATTCGCTTATTTATTTTTTACTTTCTGCCATGTTCCGCTGCCACCGTATTTCTTTT
CGGCCACTTAGTGCGCTCCACTTGATAATGATGTTTTGTTTTTCGCCGGGACAAACTCGTTTCGATTAT
GGGGAAAAGCGCGTATAAATCATCGCCGCCGAAGTCTGGCAAAACAGCAAATTGAAAACTGCAAAC
TGCAAACTGAAAACTGTAACCGAAACAAACACAGCATCCCACACGACGAGATGAAAATGAAAATAA
ATACGGACTGAGCGACTGAAAACGAGTCAATTCGATTCAAATTGCAGGTTCAACGGCTGCCGGCGAT
CGCATCATTAAGTGCGCCTTCGCTGGATACGCGGCTCTTATGCAACGAGCACACACAATTAATTAATA
AGCGTCTGGTTGTTTCGGCCTGGCTTTTGCGGACCTGCGGATCGCAATAAATTAAGGCAGCATTAGT
CGCAATTATGTGCCACATAGTTGGGCTGCTTACTTTTCTGTGGGTGAGCCGAGCGCAGAATGCGGCC
AAGGGATCGAGTTAAACCGCTTTTCCGCGGGCCAAGAGTTTTTCGCATTTTGCATAAAATCGGCAAC
GCATAAGTGGCGAAGCATTGATGAAACTGCGGGAAAAGAAGTAAAAAATATTTAAAAATATATATA
AATTTATGGCATGTTCTGTACAGAACTTATGAAGCTAATTTGAAATACTTCTTCTTAGGAAACTGTCCC
TAGGAATATTTGTTTTCCCCAGCATTGCTCAATATTTCCTCCATCTTTTTGCTTATTTCCCAGACATTTTC
TTGGCCGAAGTGTAGCTGGTGGGTCTCCAGATTAATGCAAACCACTTCGTCAGCGGAGGTCGTAAAC

GTATCTTTGCCCATTTGGCTCGTTCATTATGCGTGTGGTATAGCTTTATTTTTGCCATTTTCCCTCTTTTT
TGCACCAGCTGCAGTTGGGCCAAGAGAGTTATGCGAATCGGTGCGATTTTCGGGTTTTCGCACTCGC
TTGCGGCCATGGCCATTAGAGCATTACCCACTTAGGGCGCCCTAAAGTCCAGGTGGTCCCCAGGGAC
CACAAGAGTATTGCAACTTACGGCCAGCTGAGTGGAGTGCCGGAACGCACTTCTTTAATTTCGGCGG
TTATGTAACCTCGAGCTGAGTGTGCGATACATATGCCAAAATCACCTGCTCATAATTAGCGGAAACCA
ACTGTTTGGCCCTCGCCGGACTGTGAATCATCGGAGCTGCCCAATCGAAATCAAAGCCAAGCCAATC
GAAGCCCAGGTAATTCATATTTAATGCTGTTAGCAAAGACCTGTCCTAGATACTCTGTATAAAAGTAT
AATTATTAAGTGCATATCAGGTTTATTTATATTTATATCGTATAATATTGGTAAGTGCAGCAGCTGCTG
TGCTCTAAATTTAGAGTCATTTAAAACAAACATATTTGCCACAGAAAATGTGTGAAATAATTAAACTA
AAAGCTTTGGATGAAGTAAGAAAGCCATAAAGCCTAAAATAATATTATGAATAATCAAAGAAAATCA
GTAGATGGTAAAGTATTTCGTACCTACGTTGCATGGTATTCAATAAAGATTCAAAAATACTCTCACCC
ACTGTAAGTGAACCCAGTGTTTCGTAATTGCCTAGCACATAAATCAGCTGAATCCTAAACGTATCTGA
AGGCCAGGAGTGTCGGAGAATTCGGTGTGCCAAAAACCAAAGACCAAAGACCATACCCTTTCAAAA
CCTTATGAAAAATGGCAAGCCCGGCGAAAGGTGTTGGCCGGTCCAGGGGATTCGGGCGCCCGAGAT
ACTCGCACTTAATAAACATGCGTGAAAATCAATCAGCGAAGACAAAAGCCACGCACTGGAAGAAAC
CAAAGTGTCCGAAGTGGCCGATCCACAGGTGACCATATAGACCATAAAGTCCGCATGGTGGACCACC
ACCCGAGCCACCGAAAGCAGCCGAATGGCTGAAACCCCGAAGTTGGCGCCTTCGTTTTCGCTTCCAT
TGGCCTGCCTTCGTCTTCGGAGAAAAAAAACCTCATATAAAACGTGGCCGACATATTGTGGCCAACA
GTCGTAAGCGCGCCACGGTCCACAGAAGAGGATTAGACAGATACCTCAGAGATACCGAGATACCCA
GATACTAGCTCAAGAACCTCCTTGAGAACACTGCACCTAGAGTTCGCTGTTTTTTTTCTTTTTTTTGCCC
GAGTGTCCGGCGTGCTTGTTGAGATCGCAGAGTTGAGTGCCATGTTTCAGGACAAAGGACGCCTCCT
GGTGATCACCCTGATCGCCCTGGCCACGCCCTCGTGGGCCGCCTACAAGCTGCAGGAGCGCTACAAC
TGGAACCAGCTGGACTTCGCCTTCCCCAACGCCCGCCTCAAGGAGCAGGCGCTGGCCAGCGGGGAC
TACGTCCCCCAGAACGGCCTTCCCGTGGGCGTCGAGCACTTCGGCAGCCGGCTCTTCGTCACCGTGC
CCCGCTGGCGAGATGGTAAGTTGGTCCTCTGGCCGGGAGCAGCTCCTTTTGGGGAGCCGGGCAGAT
TGCCAGTGAGCCCGTCCCGCTTTGGGCATCATTAGCACTTACGAATTGGGCGTGGAATCTAAGTGAT
TGATTGGCCGCGGTTCACAGTTTTCGTATATGGCCAAATTCGAATTGACCAAAACCAGAGTGCGATA
CAAGTGAAAATCAGCGCAGAAATAGCGACGGATGCCGTAAACAAATTGACAGTTCGTGCGGCAAAA
ATGTGTGAGGTATTTCGGAGATATTTACCGCAGATTAGTATCTGTGCAGTTACGTAGTTGAATACCGT
GGGTATCTAAAATAGTGTGGGTCAGCTGGGTTTCTCATAGGGGTTTTACAAGACCAAGTTCTGGACT
TATAGTAGGGCATGGTGTTTATTATAGCCGGATAAAAAGATCCTTTTTTTAGAATAGAAAGATGCATT
ACAATTTGCGTTTATATTAATTATAAATAGTGTAGTATAATATATCATAATTTTAGTTAGAACATTTCT
ATTATTGTTTTATTTTCCTTGAGAAAAGGTTTAATTTTAAAATAGTTAAAAATATAAACCTTAAATTAA
ATGCTTAAATAGAACATGTGTGTTTTATGAGAGAGGAGGCTTAGCATAGCAGTCTGTTTAAAAAATT
CAATTGAATTATTTGTGGCCTAGAAAAAGTTTAAAGTTTAAAACTAAGAAGAGTTTAACAAAAGATTA
AATATGCATCCTATATTCTGGACACTTCAAGAATTAAGAATTTTTGAGTTCTGTTCCATTTTATTTTATT
TTTGAAACATTTGGTTAAAATACTAAAAGTAGGAATTTAGCCCCTAAGGAATTGTTTCTCCTTAGTTTT
TTGTGGAAACCCTAGGAGTAGCAACGAATATTTCCCAGTAAACCTACTCAAATATGTGGTAGCTCACC
CAAATATAAACCAAAAATCTATTATGATACTGGCTTCATTTCCGGCTAAGAAAGAACCCAATAGAAGT
TCCAGAAAAGTGACAAAGATTTTGATAGACGGTTTTCGGGTTTCAGTGTTCAGCGCGTTGACCTCTCA
TAAATCCGGCTGTAACTCGCGTTTGCGTGCTGCATTTACATTTGAAAACTTAAACAATTAACCGTTGT
AAAATCGGAGCCAATAGCGCGGCATTGGCATTATATGGTATTATATGACCATCCGAGTTGTGAAGGC
TTCCCCAGGGTCGCCCCACACAAATGGAAATGGAAATCCAATCACACACAATAACTTATTATGCATTT
TGCCATTAACCAATGATTAGCTTTCAGTTGGATCGAAGTGAGAGTTGCACAAAGTCTGAGTATAAAG
ACATTGTTCTGCGGCACAGAGGCTGTTTCACACACAAATTAGCAAATGGTTTTTGTTTCGCCAGCTTG
CAAAATTGCCACAAAATAATATTTCACAAGTGGAGGCAGGAAACACTGATTTATCGATGATTTGGTA
AACGTTTATGGGTTACAATACAAATTAATTTCAAGTTGGTTACTATATCTAAAACTCATACAGATTGTT

TAAGCTTGATAAATAAAAAGATAAAGGCTAGTAGTTGCGTGAAATAATGAGGTATTAAGATTACAAA
TTTCAAATAGGACCATTTCAAAGAAAACTTAATTAGTAAATGTTAGTAGTTACGAAGTACATCCCGGT
ATTCTGGCCCAACCTTGGCGTTTCTTTTGAAATAGATTTCGAACGCCTATCTCCGAGCATTAATAACAA
AAAGCCAGAAGGCCGAATCGGCAAAAAATAAAGCCCGAATTAAAGCAAACAAAAGCACGTGAAAGT
TGCGGCAAAGCGGAGTTTTTGGAGGCACGCTTCAATGGCCATCGAAGCAAATCAATTAGTCGGAGC
AAATCTGCGGTGGCAACAAGTGCTCGAACCAAATCCCCCTGAGTTGACAGTTATGAGCCATGATTAA
TGGGACTTGGGCCAATCGAAGTCCATTAGATAATCCCCCGACGAAGGTTCGTGTAAATCAGTTCCGA
TCACTAAATAAACAGACAGTTATTGCGGCATGTGTTCGCCGTCTGTTCCCACATACGAAATGAGCTGG
GCGACAGGATTACAAATTGAAATCGAGTCTGAGAAATCGAATTCGAAATTGAAACGAGAATGCGAA
TGCGATCGAGCGACTGCGACTGCGGCGAACGCCGAGAATTGCCAACTTTAATTGGCCGGATAACGA
CTCAACAGCTCGTGTTCCATACGGATTCATTAGGCTGCCATTAGCCATTGAACCACCAGCCGCCGGGC
ATCCAGCCACTTGGCCACTTGATCCACGGAGCCATTCAGCCATTGAGCCATCGAGTGGACGCACTGG
CCACCACAGCCCCTGCCTGTGTGGTCTGCGCCAAGAAGCGGCGCTATCTCAATCGCAAATGAGATCT
CAAAAAATGGCGGACTTTCACGAGGGGCGGGGACTCGATAGCTGGCTTACCAAAAAATCTATAAAA
CAAGAATGCTGATAATTCAATTACTCTTTGGGCTGTCAAGCATTTTGTTAGATGAAAAACGAGTCAGG
TAATTCCAAAGAAAAATCGTAGTTCCGAAGAAATTATCTTAGAGATACATAGATATTATGAATTATCT
ATAGGCTTAGGCTACAATTTTCTCTATAAACCTAAGATTGGAACTAACCGAAACTGTTTCATAAATATT
ATCTAGAAGTTTGGTCTATCATTTCCCTTTTAAACAAAGAAAATTCCAAAAAATACTTTTTAAATACTTT
GAAACTGCCTATAATTTTAGCATCCAAAGAATTCATACTCTTCATTTCATTGAAGATTTCCATTATAAT
AAATTAGCTATACATAATCAATTAGGTATTTATTTTCCCACCTCGCTATCAAAGCCATTAGTAACCGAT
TTCCCAAATCCCCGTCGAACAACTCAATTCATTTAAAAACACACGAAGTGAAAAGCGCCGATGGGGC
GGAAAAATCGCTGTCAGATGTATCACATAATTTTTGATTAATGGGCAGACCGAATGGGGCGGTGCG
GTCCGCCAAGGGGCGTGGCAGTCGCTCGCCCGCCACACCGAAAGAAAAAGGTAGGCAAAGTCAGA
GACCAGAAATGGACGGGAATTCAATTAACCAAGAGATATGGAAAAGAAGCAACTGGAGCGGAAAT
CGCTCGAGACACTGGCGGTGGTCGTCGCATTATATCAACAATGCATCACGGTTGTACCGCTTAGATG
CATAGATATTGATGGTGATGTCAGAGCGGGTTGCCTCAGTCTGCCGGCTAATGACATCCGAATTGCA
CGCAACTTGCAACAGTGCCGATTTATGGGCCAACTCGTGACTCAAACTCAAGTCTACCCAAGGCGAG
GCACTTCAGCCACATAGAACTCCCAGAAATTGTACAATTCCAGAGACTAAAAGTGTCGTTAGCGCGG
CGCAATGGTCTATAATTTGAGTTCCGGGTGTCGAACATGGTGGCAAAGCCATTTGCATAACACCCAA
ACGACTTCTTTCCTCTGAGAAGACAAGTCTCAGGATTTTAATTGGGTTTTTGGCGACAGAAACGAGG
GTACTTATTTTATTACCAACTCAGTTTTTATTATCTGGCGGATAGAGCACTTTTGCTAAGTGGAATCAT
AAAGTTGGCACATATTTGCATAATTTTTTATAAATTTTAAGATACATATTTGTTATTAAATGCAATGGT
TATTGAAAGTAAACCAGGTCAAGTCTTACAAATTGCAAATAAATATGTAATGATTGAGGGGCAATAA
CGATTATTCCCATTTTACTTTTGCTTTTAATGAGACAGCCTTCTATTGGAATTGGGAAACTAGTTAGTT
TTGCTTACTGTATCTCTATTTTCGTCGGTCTTCTAGGCATCCCTGCCACTCTGACTTACATCAACATGGA
CCGCACCCTGACGGGCTCGCCGGAGCTGATTCCCTACCCGGACTGGCGCTCGAACACGGCCGGGGA
CTGCGCCAACAGCATCACCACCGCCTACCGCATCAAGGTGGACGAGTGCGGGCGTCTCTGGGTGCT
GGACACCGGCACCGTGGGCATCGGCAACACCACTACCAACCCCTGCCCCTATGCCGTGAACGTCTTC
GACCTGACCACGGACACCAGGATCCGGCGCTACGAGCTGCCCGCCGTGGAGACCAACCCGAACACC
TTCATCGCCAACATCGCCGTGGACATTGGCAAGAGCTGCGACGACGCCTTCGCCTACTTCTCCGATGA
GCTGGGCTACGGCCTGATTGCCTACTCCTGGGAGCTGAACAAGTCGTGGCGCTTCTCGGCCCACTCG
TACTTCTTCCCGGACCCCCTGCGGGGCGACTTCAATGTGGCCGGCATCAACTTCCAGTGGGGCGAGG
AGGGCATCTTCGGCATGTCCCTGTCGCCCATTCGCTCGGACGGCTTCCGCACCCTGTACTTCAGTCCC
CTGGCCAGCCACCGCCAGTTCGCCGTCTCCACGCGGGTGCTGCGCGACGAGACCAGGACGGAGGAC
AGCTACCACGACTTCGTGGCTCTGGACGAGCGCGGTCCGAATGCCCACACCACCGCGCGGGTGATG
AGCGACGACGGCATCGAGCTGTTCAACCTGATCGACCAGAACGCCGTGGGCTGCTGGCACTCCTCTA
TGCCGTACTCCCCCCAGTTCCACGGCATTGTGGACCGCGACGATGTGGGCCTGGTCTTCCCGGCCGA

CGTGAAGATCGACGAGAACAAGGATGTGTGGGTGCTGTCCGACAGGATGCCCGTCTTCCTGCTCTCC
GACCTGGACTACTCCGACATCAACTTCCGCATCTACACGGCCCCGTTGGCCACCCTGATCGAGAACAC
GGTGTGCGATCTGCGGAACAATGCCTACGGACCCCCCAATGCCGTCTCGATTCCAAAGCAGCCCGCC
CTGCCCATTGGCCCGCCCTCGTACACCAAGCAGTATCGCCCTGCGCTGCCCCAGAAGCCGCAGACCA
GCTGGGTTTCGTCGCCTCCGCCTCCGCCGCCGAGCCGCACCTACCTGCCGGCCGCCAGCTCGGGAAA
CGTGGTCTCCAGCATCAGCGTGTCCTCGAACGCCGTGGGTCCCGCCGGCGTTGAGGTGCCCAAGGCC
TACATCTTCAACCAGCACAACGGCATCAACTACGAGACGAGCGGCCCCCACCTGTTCCCCACCCACCA
GCCCGTCCAGGCGGGCGCTCCGGAGGGCCTGAAGACCTACGTGAACTCCCGCCAGTCCGGCTGGTG
GCACCACCAGCACCAGGGTGAGGGTCGCGGATCGCTGCTGACCTGCGGCGACGTGGAGGAAAACC
CCGGCCCCATGGCCTCCTCCGAGGACGTCATCAAGGAGTTCATGCGCTTCAAGGTGCGCATGGAGG
GCTCCGTGAACGGCCACGAGTTCGAGATCGAGGGCGAGGGCGAGGGCCGCCCCTACGAGGGCACC
CAGACCGCCAAGCTGAAGGTGACCAAGGGCGGCCCCCTGCCCTTCGCCTGGGACATCCTGTCCCCCC
AGTTCCAGTACGGCTCCAAGGTGTACGTGAAGCACCCCGCCGACATCCCCGACTACAAGAAGCTGTC
CTTCCCCGAGGGCTTCAAGTGGGAGCGCGTGATGAACTTCGAGGACGGCGGCGTGGTGACCGTGAC
CCAGGACTCCTCCCTGCAGGACGGCTGCTTCATCTACAAGGTGAAGTTCATCGGCGTGAACTTCCCCT
CCGACGGCCCCGTAATGCAGAAGAAGACTATGGGCTGGGAGCCCTCCACCGAGCGCCTGTACCCCC
GCGACGGCGTGCTGAAGGGCGAGATCCACAAGGCCCTGAAGCTGAAGGACGGCGGCCACTACCTG
GTGGAGTTCAAGTCCATCTACATGGCCAAGAAGCCCGTGCAGCTGCCCGGCTACTACTACGTGGACT
CCAAGCTGGACATCACCTCCCACAACGAGGACTACACCATCGTGGAGCAGTACGAGCGCGCCGAGG
GCCGCCACCACCTGTTCCTGTACAAGAGCAGGCACAGAAGGCATCGCCAGCGCTCTAGGAGCCGCA
ATCGCAGCCGAAGTCGCAGCAGTGAACGAAAACGCCGTCAACGGAGCCGAAGTCGCAGCAGTGAA
CGAAGACGCTACTTGTACAGCGGCCGGCCGCGACTCTAGCCCAACCCGTGCACGGAGCATGGGCGC
CTTCACAACCACACGCTTGATGATAGGATATTTAAATAAATCACGAGGAAACGAATCTTAAACACGG
GGCAACAGCGGTTATTTAATACAAATCCCACGATGTTTTGGCCAGTGGCAATCCAAGAATTTCACCTA
GTTTTGTACTTTAAGCAAGAAATAAATAAATGATTTTTTTTATAAAGGAAGGGGATTTTGTCATAAAT
AGAAAATGAGGAGTATCTGTAAGTATGCT

>R6_1kb
TACAATTTTATAGGGTTGTGTCTACTCATGGAGTAAGGTTAGCCATTACCGACTTCTCCAGAGATACC
CAACCTTTATTAGTATTATTAGCCGTGACCTTGACATATACACACCAACTAGTATAACAAATCGGCTG
GTTGAATTCTTTTATATTAATATCTAGTAGGGCTCGAGGTTAACCGAGACAAACATAAGATACAGCTA
TTATAAAAGAGTAGGGTGGCGAAACGATAGTCCAGATTTGTCCATAAACGTTATCATGTACTGGAAA
GGTTCCCATGGGAGACATAGAGTCAGGCCTTAATATTTAAAAAACGGTACTAACTCTAGTATAAAAG
ATTCAACGGAGACCACATTAGCTCCATGCCGAGCCCTTGATTACCGACATAATTTTTATAATCAAGCC
TGTCCCGGGCACAGCGCGTATAGTTCCTTATATTATTATTTCATATTCTATATATTATATTATGCCTAAC
CTAGCGTGTACTCGGAGAGATCTTGCTCTGTGAGAGGGCCTCTAACTCACATTAATTATAAATGTAGC
CCGATGTTCCTTAAATTAATTAGTATCCTGCGCCGAAGAGTGGATAACCAATCTTATAACAACTGTGG
TGTATGATTTGGAGCTAGAGCTACTGTCAATGTATATTTAACGTTAAAGGACCCCCATACGCTTTTCA
TCTGACCAAACAACGATCTTGGACTTAGTAATTGGCTCATGACAATTATAGATTGGATAATCCGACAC
TATTCCGACGCCTATCCGCGACAGGTAGGCGAGACCTAATGCCTATATTATTATTCGGTTAATTATCT
GGGATATTAAGGCGGATAATTATAAACCAGTCTGCAGCGTTTCAAAAGGACTAGTCGACGCTTTCAT
GTGTGAAGGATAATAAGTCAGGAAGCTTATTATTAATAATGATGTAGTGATGTAACGGCGGTTGTTT
TTGACAATAAGGTACTCAACATCCTACGTAAGAGTTCCTTGTCCAAGTGGCGTAA

>R6_500bp

TACAATTTTATAGGGTTGTGTCTACTCATGGAGTAAGGTTAGCCATTACCGACTTCTCCAGAGATACC
CAACCTTTATTAGTATTATTAGCCGTGACCTTGACATATACACACCAACTAGTATAACAAATCGGCTG
GTTGAATTCTTTTATATTAATATCTAGTAGGGCTCGAGGTTAACCGAGACAAACATAAGATACAGCTA
TTATAAAAGAGTAGGGTGGCGAAACGATAGTCCAGATTTGTCCATAAACGTTATCATGTACTGGAAA
GGTTCCCATGGGAGACATAGAGTCAGGCCTTAATATTTAAAAAACGGTACTAACTCTAGTATAAAAG
ATTCAACGGAGACCACATTAGCTCCATGCCGAGCCCTTGATTACCGACATAATTTTTATAATCAAGCC
TGTCCCGGGCACAGCGCGTATAGTTCCTTATATTATTATTTCATATTCTATATATTATATTATGCCTAAC
CTAGCGTGTACTCGGAGAGATCT

R1 probe set (87 oligos) :
>reporter1:1-1980:0-45
GTCAGACGCAGAATAATAATAATTAAGCAATACGTGTCACCCGAA
>reporter1:1-1980:43-88
AATGACGACCCCTATACATCGCTCTCCAGAGTATAATATTAAAGG
>reporter1:1-1980:86-131
GGCTTCATGGCGTTATAGTAGTTGACTGGTTACTGAGACTATCGC
>reporter1:1-1980:129-174
GCATATAGGTAGGTAAAGCGTCATTGTAATAGTACGGTTTAGCTT
>reporter1:1-1980:175-220
ATCACTTTTTATCAATCTCAAGTTTCTCGCCGTTTAGTAGTTCGA
>reporter1:1-1980:218-263
GACACGTTAATTAAGCGTATAACGGCATCAGTCTACACGCTACCC
>reporter1:1-1980:261-306
CCGTATTCGGGACCGCGCGAAGAATTATTACTCTTTTATATATTC
>reporter1:1-1980:304-349
TCAAGGAAGTGAGTGATGTAAAATATATTTACGTAGGTTCTCGAC
>reporter1:1-1980:347-392
ACCGCGGCCCATCGTTACCACATAAAGGTATATTAAATAGACGAC
>reporter1:1-1980:390-435
ACCCCGCGCCCGTAGGGATCATGCCTTATTATATAACTTAGCTGT
>reporter1:1-1980:433-478
GTAGGCTATTTTGACGTTAATCGGTCAGTACTGACTAGGTACTAG
>reporter1:1-1980:476-521
AGCAATCTTCGACGTATATGGCTCTACGACGTCTTCAAAATTATA
>reporter1:1-1980:519-564
TATATTACGGTAGAGGGCCAAGCTCACATGAGATTATTATATTTT
>reporter1:1-1980:562-607
TTACTTCGCGCCCCTAGAAACAACAAATTATATGACGGTTAAAAG
>reporter1:1-1980:620-665
TCCAGCGTTGGAATGCCCGGCGGCTGTATCTATATATATTAAATT
>reporter1:1-1980:663-708
TTAACGGTCTAGAGCGAACCATTTCTTCCTATCTAAAACCGCGAT
>reporter1:1-1980:706-751
ATACAAGTCTGGCCCTCTATTAATCTTTAACTCTTCCGGGTAAGA
>reporter1:1-1980:749-794
GATTAGGATAACGGACGGTTTGAGTTTGTACCAGTAATCGGAGTG
>reporter1:1-1980:792-837
TGCGCTTAAATTTACGATGATGTTATAAAAAATCAGATCTCAACG
>reporter1:1-1980:848-893
GATGCGATAGAGCTTGGGCCTCACATCCATATTATTTATATTGTA
>reporter1:1-1980:891-936
TATAGAGGCAAACACTATTAATCTTAATTCAGATCTGGGGCGCCG
>reporter1:1-1980:934-979
CGGTAGTTGATCCTATGACCTTCAGCAACGGTTTACTGAAGTTCC
>reporter1:1-1980:977-1022
CCTTATAACTCGGGCCGATTTCACGTGAATGTAATAATAATAATA

>reporter1:1-1980:1020-1065
TATAACGCCCTATGGGTATTACCCATAAGGGCGCGCTTGATTAAC
>reporter1:1-1980:1063-1108
ACACATAACTTTACCACACCACAAAACGGAGAAGGTACAATATTA
>reporter1:1-1980:1106-1151
TATTATTAAGGGATGCCTATCCCTACACGAAAACTTTTAATATTT
>reporter1:1-1980:1149-1194
TTTAATCCCCGGCATGAATCGATCTCAGGGGGCTTTAAAATTAGA
>reporter1:1-1980:1192-1237
GATATTACAGAGTCGGGGACTATTCCTTCTTATAAAATATAGTCG
>reporter1:1-1980:1235-1280
CGATATAAAGTGCCGTATAGGGGCTGGAAGATAATTATCTTTTAC
>reporter1:1-1980:1278-1322
ACCTGCGCAGTTAAGGATAACGGTTGGCAAGTCACATGAACTCG
>reporter1:1-1980:1320-1367
cgtctgatccaatttataagaaaaagtcacaacgaatataatataat
>reporter1:1-1980:1381-1425
GCCCCCCGACCATGAAGTTTCCATATTATAAGGGACGTAGCACC
>reporter1:1-1980:1423-1468
CCCGGCTCAGTAATCCATTTGGCCTTGTGTAATATTACGATTCAG
>reporter1:1-1980:1466-1511
AGCGCACAGCAAATGACAGATGTAAGCTAGCCCCTCGTGAATAAC
>reporter1:1-1980:1509-1554
ACTGAAACTAGTTGTTCACCACAAATGGGCCTCAATAGGCTCATA
>reporter1:1-1980:1552-1599
tatagttcagtgatacattccaaattaattaatataaatcaatgcta
>reporter1:1-1980:1616-1661
AGGACCTCCCCAGGGTTGGCTCTTAACTCGGTCACTAAATATTTA
>reporter1:1-1980:1662-1707
TTCTTATATTTTCAGCGGAAGGTTGCGTGATATTTTAACACGAAG
>reporter1:1-1980:1705-1750
AGACTAGGACTGAGTCATTAATAAAACTATCAGTAAGAGACCGTA
>reporter1:1-1980:1748-1793
TACACAGCGTGTCAGGAATAGGACGCTACCAATAGTTGATAAAAC
>reporter1:1-1980:1797-1842
TAAGGGACTGTCCCCACCCAGCCCTGCAATATATATTAATCTTTA
>reporter1:1-1980:1840-1887
taaacgagcgataatttacctataagaacttataacttgtgctatag
>reporter1:1-1980:1885-1930
AGTCCCACAAGCCTATTGATAGTAAGTGGAGTATCGAAGTATTAG
>reporter1:1-1980:1928-1973
AGATTCCTGTCGAGCCAATTTTAGAAGTACTTCCAACGTTCTCTG
>reporter1:1-1980:22-67-rev
GAGCGATGTATAGGGGTCGTCATTCGGGTGACACGTATTGCTTAA
>reporter1:1-1980:65-110-rev
CAACTACTATAACGCCATGAAGCCTTTAATATTATACTCTGGAGA
>reporter1:1-1980:108-153-rev

ATGACGCTTTACCTACCTATATGCGATAGTCTCAGTAACCAGTCA
>reporter1:1-1980:151-196-rev
TTGAGATTGATAAAAAGTGATGAAGCTAAACCGTACTATTACAAT
>reporter1:1-1980:197-242-rev
CGTTATACGCTTAATTAACGTGTCGAACTACTAAACGGCGAGAAA
>reporter1:1-1980:240-283-rev
cttcgcgcggtcccgaatacgggtagcgtgtagactgatgccg
>reporter1:1-1980:283-330-rev
atattttacatcactcacttccttgaatatataaaagagtaataatt
>reporter1:1-1980:326-371-rev
TATGTGGTAACGATGGGCCGCGGTCGAGAACCTACGTAAATATAT
>reporter1:1-1980:369-414-rev
GCATGATCCCTACGGGCGCGGGGTCGTCTATTTAATATACCTTTA
>reporter1:1-1980:412-457-rev
CCGATTAACGTCAAAATAGCCTACAGCTAAGTTATATAATAAGGC
>reporter1:1-1980:455-500-rev
GAGCCATATACGTCGAAGATTGCTAGTACCTAGTCAGTACTGACC
>reporter1:1-1980:498-543-rev
AGCTTGGCCCTCTACCGTAATATATAATTTTGAAGACGTCGTAGA
>reporter1:1-1980:541-586-rev
GTTGTTTCTAGGGGCGCGAAGTAAAATATAATAATCTCATGTGAG
>reporter1:1-1980:584-629-rev
AACGCTGGAGCCATAGTATTCCCTTTTAACCGTCATATAATTTGT
>reporter1:1-1980:642-688-rev
GAAATGGTTCGCTCTAGACCGTTAATTTAATATATATAGATACAGC
>reporter1:1-1980:685-730-rev
ATTAATAGAGGGCCAGACTTGTATCGCGGTTTTAGATAGGAAGAA
>reporter1:1-1980:728-773-rev
CTCAAACCGTCCGTTATCCTAATCTTACCCGGAAGAGTTAAAGAT
>reporter1:1-1980:771-816-rev
AACATCATCGTAAATTTAAGCGCACTCCGATTACTGGTACAAACT
>reporter1:1-1980:814-859-rev
TCTATCGCATCGTGAGTAAAGCCGTTGAGATCTGATTTTTTATAA
>reporter1:1-1980:870-917-rev
taagattaatagtgtttgcctctatacaatataaataatatggatgt
>reporter1:1-1980:913-958-rev
TGAAGGTCATAGGATCAACTACCGGCGCCCCAGATCTGAATTAAG
>reporter1:1-1980:956-1001-rev
GTGAAATCGGCCCGAGTTATAAGGAACTTCAGTAAACCGTTGCTG
>reporter1:1-1980:999-1044-rev
GGGTAATACCCATAGGGCGTTATATTATTATTATTACATTCACGT
>reporter1:1-1980:1042-1087-rev
TTGTGGTGTGGTAAAGTTATGTGTTAATCAAGCGCGCCCTTATGG
>reporter1:1-1980:1085-1130-rev
AGGGATAGGCATCCCTTAATAATAATATTGTACCTTCTCCGTTTT
>reporter1:1-1980:1128-1173-rev
GATCGATTCATGCCGGGGATTAAAATATTAAAAGTTTTCGTGTAG

>reporter1:1-1980:1171-1216-rev
AATAGTCCCCGACTCTGTAATATCTAATTTTAAAGCCCCCTGAGA
>reporter1:1-1980:1214-1259-rev
GCCCCTATACGGCACTTTATATCGACTATATTTTATAAGAAGGAA
>reporter1:1-1980:1257-1302-rev
ACCGTTATCCTTAACTGCGCAGGTAAAAGATAATTATCTTCCAGC
>reporter1:1-1980:1300-1345-rev
TTTTTCTTATAAATTGGATCAGACGAGTTCATGTGACTTGCCAAC
>reporter1:1-1980:1343-1388-rev
GGGGGGCCGACGTTTATTTTAATTATATTATATTCGTTGTGACTT
>reporter1:1-1980:1403-1448-rev
AGGCCAAATGGATTACTGAGCCGGGTGCTACGTCCCTTATAATAT
>reporter1:1-1980:1445-1490-rev
TACATCTGTCATTTGCTGTGCGCTGAATCGTAATATTACACAAGG
>reporter1:1-1980:1488-1533-rev
TTGTGGTGAACAACTAGTTTCAGTTATTCACGAGGGGCTAGCTTA
>reporter1:1-1980:1531-1576-rev
TTTGGAATGTATCACTGAACTATATGAGCCTATTGAGGCCCATTT
>reporter1:1-1980:1575-1620-rev
TCCTGGGGAAGCCACTGCGCCTAGCATTGATTTATATTAATTAAT
>reporter1:1-1980:1638-1684-rev
CCTTCCGCTGAAAATATAAGAATTAAATATTTAGTGACCGAGTTAA
>reporter1:1-1980:1684-1729-rev
TTATTAATGACTCAGTCCTAGTCTTCGTGTTAAAATATCACGCAA
>reporter1:1-1980:1727-1772-rev
GTCCTATTCCTGACACGCTGTGTACGGTCTCTTACTGATAGTTTT
>reporter1:1-1980:1770-1815-rev
GGTGGGGACAGTCCCTTACGATGTTTTATCAACTATTGGTAGCGT
>reporter1:1-1980:1819-1866-rev
cttataggtaaattatcgctcgtttaaagattaatatatattgcagg
>reporter1:1-1980:1863-1908-rev
ACTATCAATAGGCTTGTGGGACTATAGCACAAGTTATAAGTTCTT
>reporter1:1-1980:1907-1952-rev
CTAAAATTGGCTCGACAGGAATCTAATACTTCGATACTCCACTTA

R6 probe set (87 oligos) :
>reporter6:1-1980:0-45
TACAATTTTATAGGGTTGTGTCTACTCATGGAGTAAGGTTAGCCA
>reporter6:1-1980:43-88
CATTACCGACTTCTCCAGAGATACCCAACCTTTATTAGTATTATT
>reporter6:1-1980:86-131
TTAGCCGTGACCTTGACATATACACACCAACTAGTATAACAAATC
>reporter6:1-1980:129-174
TCGGCTGGTTGAATTCTTTTATATTAATATCTAGTAGGGCTCGAG
>reporter6:1-1980:172-217
AGGTTAACCGAGACAAACATAAGATACAGCTATTATAAAAGAGTA
>reporter6:1-1980:218-263
GGTGGCGAAACGATAGTCCAGATTTGTCCATAAACGTTATCATGT
>reporter6:1-1980:261-306
GTACTGGAAAGGTTCCCATGGGAGACATAGAGTCAGGCCTTAATA
>reporter6:1-1980:304-351
TATTTAAAAAACGGTACTAACTCTAGTATAAAAGATTCAACGGAGAC
>reporter6:1-1980:358-403
GCTCCATGCCGAGCCCTTGATTACCGACATAATTTTTATAATCAA
>reporter6:1-1980:401-446
AAGCCTGTCCCGGGCACAGCGCGTATAGTTCCTTATATTATTATT
>reporter6:1-1980:444-491
TTTCATATTCTATATATTATATTATGCCTAACCTAGCGTGTACTCGG
>reporter6:1-1980:489-534
GGAGAGATCTTGCTCTGTGAGAGGGCCTCTAACTCACATTAATTA
>reporter6:1-1980:532-577
TATAAATGTAGCCCGATGTTCCTTAAATTAATTAGTATCCTGCGC
>reporter6:1-1980:575-620
GCCGAAGAGTGGATAACCAATCTTATAACAACTGTGGTGTATGAT
>reporter6:1-1980:618-663
ATTTGGAGCTAGAGCTACTGTCAATGTATATTTAACGTTAAAGGA
>reporter6:1-1980:661-706
GACCCCCATACGCTTTTCATCTGACCAAACAACGATCTTGGACTT
>reporter6:1-1980:704-749
TTAGTAATTGGCTCATGACAATTATAGATTGGATAATCCGACACT
>reporter6:1-1980:758-803
CCTATCCGCGACAGGTAGGCGAGACCTAATGCCTATATTATTATT
>reporter6:1-1980:801-846
TTCGGTTAATTATCTGGGATATTAAGGCGGATAATTATAAACCAG
>reporter6:1-1980:844-889
AGTCTGCAGCGTTTCAAAAGGACTAGTCGACGCTTTCATGTGTGA
>reporter6:1-1980:887-934
gaaggataataagtcaggaagcttattattaataatgatgtagtgat
>reporter6:1-1980:932-977
ATGTAACGGCGGTTGTTTTTGACAATAAGGTACTCAACATCCTAC
>reporter6:1-1980:975-1020
ACGTAAGAGTTCCTTGTCCAAGTGGCGTAATATTATTACGTCATA

>reporter6:1-1980:1018-1063
TAACAGCTAATCTAACACATGATGTGCAAAAGGGTTATGACTGTC
>reporter6:1-1980:1061-1106
TCCTAGAACCAATGCCCCAAAGTCTGCGTTACTCTAGGACAATTT
>reporter6:1-1980:1104-1151
ttaacattttagacgtgccatataatgcttatatatttaagctatgt
>reporter6:1-1980:1149-1194
GTATCGAGAGCAGGGACCCCAGTTAAATATTTATATATGTTAGAC
>reporter6:1-1980:1192-1237
ACCCTTCCGATGAAGTAGGTTGGACTATTTTAATGATTTCATATG
>reporter6:1-1980:1235-1278
tgtaacgaccgctgtaactacgggatgatcggctgctagctca
>reporter6:1-1980:1276-1323
caggtaatatatttaacgataacctgtctttccgtaatataattatt
>reporter6:1-1980:1339-1384
AACGGGATCGGCAGAAATGTGGATTCTTACGTGATTCTGAAGTAA
>reporter6:1-1980:1382-1427
AATGTGTCGCGGAAAATATTATAATAATCCAGACCCGTTATTCTC
>reporter6:1-1980:1425-1470
TCGTAGCTACGCCCTAAAGCGAATAATATGTTTTATACATTAGAT
>reporter6:1-1980:1477-1522
CCGCCGAGTTGTCTGACGCACCTCCGTTAAAAATATATTGTAAAT
>reporter6:1-1980:1520-1565
ATGAACCGTTCCTCTTAGCATGTAGAGTTTTCCTGATAATAAAGT
>reporter6:1-1980:1563-1609
GTCTCAGATAGAGTATCCTCCGGATTAATATAATAATTTCAATCCA
>reporter6:1-1980:1613-1658
CTTGGGTCAGCCCTTGGCCAGTCTGTGGAAATAATATAGAGAACG
>reporter6:1-1980:1656-1701
CGTTTCTAGTGCCGGATTATCTTAAGGGGCGGTTAATTAAATATT
>reporter6:1-1980:1699-1744
TTCTGATAGACATCGAGTTGGTCCCTTATTAGTGTACGGCTTTTG
>reporter6:1-1980:1742-1787
TGTCTCCTGATTAAACTGAGACTCGTATATCTTTTATGAGTAGAC
>reporter6:1-1980:1785-1830
ACCGCGTCGTTAGAAGAGTTAGTTATATTATTTATGCCCTACTGT
>reporter6:1-1980:1836-1879
tgcccgctccctattcgtagtataattctaggtgcgccctcgg
>reporter6:1-1980:1877-1924
ggttataatttttgtagctttagttatattatatcatagccgacttg
>reporter6:1-1980:1931-1976
CTCAGCGGTTCACCAGATGGATGTTTAATATATAGAGAACCGGTG
>reporter6:1-1980:22-67-rev
GTATCTCTGGAGAAGTCGGTAATGGCTAACCTTACTCCATGAGTA
>reporter6:1-1980:65-110-rev
TGTATATGTCAAGGTCACGGCTAATAATACTAATAAAGGTTGGGT
>reporter6:1-1980:108-153-rev

ATATAAAAGAATTCAACCAGCCGATTTGTTATACTAGTTGGTGTG
>reporter6:1-1980:151-196-rev
TCTTATGTTTGTCTCGGTTAACCTCGAGCCCTACTAGATATTAAT
>reporter6:1-1980:194-239-rev
CTGGACTATCGTTTCGCCACCCTACTCTTTTATAATAGCTGTATC
>reporter6:1-1980:240-285-rev
CTCCCATGGGAACCTTTCCAGTACATGATAACGTTTATGGACAAA
>reporter6:1-1980:283-328-rev
AGAGTTAGTACCGTTTTTTAAATATTAAGGCCTGACTCTATGTCT
>reporter6:1-1980:327-372-rev
GCTCGGCATGGAGCTAATGTGGTCTCCGTTGAATCTTTTATACTA
>reporter6:1-1980:380-423-rev
GCGCTGTGCCCGGGACAGGCTTGATTATAAAAATTATGTCGGT
>reporter6:1-1980:423-470-rev
cataatataatatatagaatatgaaataataatataaggaactatac
>reporter6:1-1980:467-512-rev
CTCTCACAGAGCAAGATCTCTCCGAGTACACGCTAGGTTAGGCAT
>reporter6:1-1980:511-556-rev
AAGGAACATCGGGCTACATTTATAATTAATGTGAGTTAGAGGCCC
>reporter6:1-1980:554-599-rev
AAGATTGGTTATCCACTCTTCGGCGCAGGATACTAATTAATTTAA
>reporter6:1-1980:597-642-rev
TTGACAGTAGCTCTAGCTCCAAATCATACACCACAGTTGTTATAA
>reporter6:1-1980:640-685-rev
TCAGATGAAAAGCGTATGGGGGTCCTTTAACGTTAAATATACATT
>reporter6:1-1980:683-728-rev
TAATTGTCATGAGCCAATTACTAAGTCCAAGATCGTTGTTTGGTC
>reporter6:1-1980:726-771-rev
TGTCGCGGATAGGCGTCGGAATAGTGTCGGATTATCCAATCTATA
>reporter6:1-1980:780-827-rev
cttaatatcccagataattaaccgaataataatataggcattaggtc
>reporter6:1-1980:823-868-rev
AGTCCTTTTGAAACGCTGCAGACTGGTTTATAATTATCCGCCTTA
>reporter6:1-1980:866-911-rev
AAGCTTCCTGACTTATTATCCTTCACACATGAAAGCGTCGACTAG
>reporter6:1-1980:910-955-rev
GTCAAAAACAACCGCCGTTACATCACTACATCATTATTAATAATA
>reporter6:1-1980:954-999-rev
CACTTGGACAAGGAACTCTTACGTAGGATGTTGAGTACCTTATTG
>reporter6:1-1980:997-1042-rev
CATCATGTGTTAGATTAGCTGTTATGACGTAATAATATTACGCCA
>reporter6:1-1980:1040-1085-rev
GACTTTGGGGCATTGGTTCTAGGACAGTCATAACCCTTTTGCACA
>reporter6:1-1980:1083-1128-rev
TATATGGCACGTCTAAAATGTTAAATTGTCCTAGAGTAACGCAGA
>reporter6:1-1980:1127-1172-rev
ACTGGGGTCCCTGCTCTCGATACATAGCTTAAATATATAAGCATT

>reporter6:1-1980:1171-1216-rev
TCCAACCTACTTCATCGGAAGGGTCTAACATATATAAATATTTAA
>reporter6:1-1980:1214-1259-rev
CCCGTAGTTACAGCGGTCGTTACATATGAAATCATTAAAATAGTC
>reporter6:1-1980:1256-1301-rev
AGGTTATCGTTAAATATATTACCTGAGCTAGCAGCCGATCATCCC
>reporter6:1-1980:1299-1344-rev
CCGTTATCATAGACGACTGCAAATAATTATATTACGGAAAGACAG
>reporter6:1-1980:1361-1406-rev
TTATAATATTTTCCGCGACACATTACTTCAGAATCACGTAAGAAT
>reporter6:1-1980:1404-1449-rev
ATTCGCTTTAGGGCGTAGCTACGAGAATAACGGGTCTGGATTATT
>reporter6:1-1980:1447-1492-rev
CAGACAACTCGGCGGTCACGCCATCTAATGTATAAAACATATTAT
>reporter6:1-1980:1499-1544-rev
TACATGCTAAGAGGAACGGTTCATTTACAATATATTTTTAACGGA
>reporter6:1-1980:1542-1587-rev
TCCGGAGGATACTCTATCTGAGACTTTATTATCAGGAAAACTCTA
>reporter6:1-1980:1586-1631-rev
GCCAAGGGCTGACCCAAGGCCATGGATTGAAATTATTATATTAAT
>reporter6:1-1980:1635-1680-rev
TAAGATAATCCGGCACTAGAAACGTTCTCTATATTATTTCCACAG
>reporter6:1-1980:1678-1723-rev
GGACCAACTCGATGTCTATCAGAATATTTAATTAACCGCCCCTTA
>reporter6:1-1980:1721-1766-rev
GAGTCTCAGTTTAATCAGGAGACAAAAGCCGTACACTAATAAGGG
>reporter6:1-1980:1764-1809-rev
AACTAACTCTTCTAACGACGCGGTCTACTCATAAAAGATATACGA
>reporter6:1-1980:1807-1852-rev
GAATAGGGAGCGGGCAGTTTAGACAGTAGGGCATAAATAATATAA
>reporter6:1-1980:1857-1902-rev
AACTAAAGCTACAAAAATTATAACCGAGGGCGCACCTAGAATTAT
>reporter6:1-1980:1900-1945-rev
GGTGAACCGCTGAGCCTTTCCCAAGTCGGCTATGATATAATATAA

X2 probe set (D. melanogaster)
>X:6760094-6770369:32-77
GGCGTGTGCGAATAGACTTCCAGGATACTTTGTCCAAGGTTCAGG
>X:6760094-6770369:137-182
CGCCCACCAAACTCGGTTATCCATCATATCCACTCCTTCGTTTCA
>X:6760094-6770369:180-225
CAGGGACGTATCGATGAGCTCTCTTAACAAGATCGAGGTGATCAT
>X:6760094-6770369:260-305
GAGTATGCGCCACAGCTATGACCACATCTTCACGTTCTAACCCTA
>X:6760094-6770369:303-348
TAATTTTCGGTCTGTAGAGGATCGAAATTGACACTGGCGAAATCA
>X:6760094-6770369:346-391
CATTGATATCACTGAAAACACTGAGTATGATTCCACTGCGTCGAG
>X:6760094-6770369:410-453
caaccaccagctcaaagagcgaagtcctcagataagggtggcc
>X:6760094-6770369:470-513
CTGGCTGGCAGGTTAAATGCTTCACTTAACCCCGCATCAGATG
>X:6760094-6770369:511-556
TGCTACCAAGGACCATCTATCGACACCAAAGCAACTAGAATTGGA
>X:6760094-6770369:554-599
GAGCGCAACTCTAAAGTCTCTTGCTCAACTTCAAAAATAAAGACT
>X:6760094-6770369:597-642
CTTCTTCCATTTCTGCCAAGATTTCAGTTTTTCCAGCCACTTCAA
>X:6760094-6770369:716-761
GCCGCCCTTCAAGCTATAGTTCAAGATACGAAAACACCAACCATT
>X:6760094-6770369:804-847
gtggcagtcgtggatttctggctgtcatgcagaaggccttaat
>X:6760094-6770369:861-906
AGCGAGCTAGCGAACAGAAAACTGATAAAGAAACTAACGGTGTAA
>X:6760094-6770369:926-969
tcgcggcgatcttatacaacatcgtcacagtcaacctgccgtt
>X:6760094-6770369:967-1012
TTCTTCAGAAATATCGGTAAGAGCAGAAAACCCAGATTTTAAGCG
>X:6760094-6770369:1058-1103
CCAGGGCAATCCAAAAAAGACTTGCCCATATCCTTGTCGGTACAG
>X:6760094-6770369:1171-1214
gctggcggccttgaaatatggactaggaacaacggtaccaagc
>X:6760094-6770369:1247-1292
CGCGGTAGAAAAGGAATAAACCTGCCTGAAACTAACAACAATAAA
>X:6760094-6770369:1372-1415
ggccggtacgtgggcctcattttcaaatgttccccaggaaagc
>X:6760094-6770369:1472-1515
GCCTTTGGGGATGCTTTGGCACATGAACCAAGGTCCTTCTATG
>X:6760094-6770369:1532-1575
ccccgagaccctcgtatctggaagagcaagacttcccaacagc
>X:6760094-6770369:1592-1635
ccacaggcacaaattcctccgtattccagtgacccgcgtcgtt

>X:6760094-6770369:1633-1678
TTCTATAAGCACTTACAGCGGTTTCGAAGAGTCAGCCAATAGAGG
>X:6760094-6770369:1700-1745
AATAATCATAATCAGAATCAGAATGTTACCGCCAATGGAAGTGCA
>X:6760094-6770369:1753-1798
GAACTGGAGTGGCAATCCATATGGAAACGGTCCAAACCCAAGACC
>X:6760094-6770369:1796-1841
CCGCCCTTTCCTAGCAATCAAAACGGGAATGAATATGCTGGTGGT
>X:6760094-6770369:2026-2069
taaggctgcggcggaggtacgccaattagaaacggaagtttcg
>X:6760094-6770369:2067-2112
CGCGGGAGATGGAAGCCCAAGAAAAAAATAAACAGCAAAAGGAAA
>X:6760094-6770369:2131-2174
ggagaagtcgacgatcgcagtgactcaggttccggaattggac
>X:6760094-6770369:2172-2216
ACACCTCCTACCGCAACGTTAATCTGGGGGTGCTAAACAAGAAG
>X:6760094-6770369:2247-2292
CACCGGCAACAACAATAACCTCAACAAGTCCAGTCAATGGTAATG
>X:6760094-6770369:2334-2379
GCTGTGATGCCAACCAGGACAAAGATACTTATAAGAATAAAGATA
>X:6760094-6770369:2377-2422
TAGGTATTTAAATAAGGCTAAGGCTAAAGACAAGGTAGATAAGGG
>X:6760094-6770369:2420-2465
GGCAATGAGGTGTCGGAGAACAATCTGGATAAGTCTGAGAAGCTT
>X:6760094-6770369:2463-2508
TTGAAAAATCGCAGGATAAGAAGGCAAATGACAAGGAGAACAAGT
>X:6760094-6770369:2506-2551
GTCCGACAAAAAGGAGAAGAAGAGACTGAACAGGGAGCCTGAAAA
>X:6760094-6770369:2549-2594
AAGAAATCAAAGGTTGAGAACCCCCTCGAGATTGTGGACTCGAAT
>X:6760094-6770369:2596-2641
CGTGGTCAGTGAGGAAAGCTCGGAAAATACAGACAATGTGGAAAA
>X:6760094-6770369:2668-2712
GTCTCCAGTTCCAGAGCTAGCTACCAGCACTCAGGACAGTCAAC
>X:6760094-6770369:2712-2757
AGGACCAGTCAGTGAGTGAAGAGTTGGACATCCTTGCCAAAAACC
>X:6760094-6770369:2756-2801
CGTAGAATGTCCGGAACTAGAATAAAGACTCCCATTTCGTCTACT
>X:6760094-6770369:2820-2865
GACGGGCAGATGATGATGTTGAGGACAAGTTGGAAAATCCGACTA
>X:6760094-6770369:2876-2921
GCAAAGTGGGAGGCAAAGCCTGACAAGGAAAAATCGGAGGATGAT
>X:6760094-6770369:2919-2964
ATACCATCGACAAGATTAAATCCATGAAAGTAACTAAATTCGCTG
>X:6760094-6770369:2962-3007
TGATGTCGAGATGAAGGTTACAGAAGAAAGCCAGAGTGCTGAGGA
>X:6760094-6770369:3044-3089

GAGGAAGGTACTGAGCATAAAAAGAGTACTGAAGAGAAGGATAAG
>X:6760094-6770369:3087-3132
AGCCGCCAAAAATCTCCAAGATAAAAATTGTCCTTACTCCCATTG
>X:6760094-6770369:3130-3175
TGCCCATACAACACAAGTGGTTCGTCCTAATGATGGCTTCAAGAA
>X:6760094-6770369:3173-3218
AACAATCAAGAAAAGATCTTGGACAACATGGCAACTGATGAGCAC
>X:6760094-6770369:3229-3272
ggtccccgggcccccagctcaattcctacgccggattatgcag
>X:6760094-6770369:3301-3346
GAAGCCGATGGTGGACAAGGATAAGATTGCTTCTTCCAGTTTTAC
>X:6760094-6770369:3387-3432
CCCGAAACCTGGCCATCATTTTCGAAAAAACTAGTGACAACTGCA
>X:6760094-6770369:3557-3600
gccaccgagggtaaagctaccagggcaaagatttctgtccctg
>X:6760094-6770369:3598-3643
TGCAAAAGATTCAACCCGTGCTGATGATGATCCTATTCCCACTCC
>X:6760094-6770369:3641-3686
CCAAATCTTAAGCGGAAAAGGCAAGCTATTCACAAGGAAACAGAG
>X:6760094-6770369:3684-3729
AGGATGATGTGGAGATGAAGCCTAAGAAGGCTCGATTGGAAGCAC
>X:6760094-6770369:3754-3799
GCCTGATGAACAGCAGGTAGAGAACAACGTGGAAGTCACCCAAAA
>X:6760094-6770369:3797-3842
AAGGAGGTGGAAGCAATATCCTCAGAGCCACTTCTCTCTTCTGAG
>X:6760094-6770369:3852-3895
cacgaaagcctcgcacgaaaccgcgaaaaaacgagctggacaa
>X:6760094-6770369:4001-4044
agtagccagcattccaggacgtcctctgtatcgcgaaccgata
>X:6760094-6770369:4042-4087
TAGCATATCCACCGTATCGGATATTAGTTCCATAATCGTCAGGAA
>X:6760094-6770369:4091-4134
gcgcgaaggggtagaggcatcagatcatccgaaaatggcatca
>X:6760094-6770369:4132-4177
CAACCGTGCCACGTTTAATGCATCCTTGAATGCAAAAAAACCAAA
>X:6760094-6770369:4180-4225
GTGCCGTGTTAGAATAAAGCGATGTGCTGCATTGATGGAGATGAT
>X:6760094-6770369:4254-4299
AGAAGGAGCAGAAGAATAAAGAACCGAAGAAGAAGAAAGTGGGTG
>X:6760094-6770369:4305-4350
AAAAGCCATTGAAAAGTAAGCCGAAAAGAGAGAATAGCGTTATTC
>X:6760094-6770369:4358-4403
AATCCCGAATGGCACTCCATTTCGAAGGCTGTTATCAAGTGTGTC
>X:6760094-6770369:4426-4471
GAGCCCACTCTCTCATTATATGATGTGCCATAAGGAGCACTATGC
>X:6760094-6770369:4585-4628
ccagaagccactgctactctgccaaaaaggcatgatcgagcac

>X:6760094-6770369:4626-4671
ACTTGATCGGCCATATGGGCGAGTCTCGTTTTTACTGCTCCAACT
>X:6760094-6770369:4725-4768
ggccaggtgcgaagcctttaagtagcaaaaccgtctgcctacc
>X:6760094-6770369:4784-4829
TGCCACATCTGCCAGTTTATGCAGTACAGCAAGGAAAATATGGAC
>X:6760094-6770369:4827-4872
ACCGGCATCTTACTGTTCAGCATGGCCTAACGAAGGAGGAACTAG
>X:6760094-6770369:4890-4935
AGTTGATGCTCTGCGACACAACAGACGTACCATATGCAGATTCGA
>X:6760094-6770369:4942-4985
tggcagcgccgttgggcctgaaattcaaaaaaacaaaccgaag
>X:6760094-6770369:4983-5028
AGTCGAAAGCAAGTGCAGCACTTTCAAATACAACCAAAAAAAATA
>X:6760094-6770369:5026-5071
TAAACAGCAAAAGAAGACAAACAACAGTCGCTTCATGAAAATGGT
>X:6760094-6770369:5069-5114
GTCAAAAAATCCGTAAGTCTATTGAAGCGAGAAAGAGAAGAGCAG
>X:6760094-6770369:5112-5155
aggatgaccagaacatgacccaggctaacgaagggtcggaagt
>X:6760094-6770369:5173-5218
TCCGCCAGAAATTGAGCCCTTGTTTGTGGTCAATGAGTGTCTAAT
>X:6760094-6770369:5231-5276
GACACGGACATGGAAGAAGTCTTGGAACAGCCCGTTCAACATATG
>X:6760094-6770369:5274-5319
TGAGCTTAATGGTAGACGAAAAGCCTGTGACGCTACTCAGTGGGG
>X:6760094-6770369:5345-5388
cctgatcccgagcctgttgttccatctgcacaagatgatggca
>X:6760094-6770369:5414-5457
gacgtggaggcagtagtggattcccttcagtcacacactgacc
>X:6760094-6770369:5455-5500
CCAGACGGCTACTTCTATGTTAGCAGAAGTCAGTCTAGCCGAATT
>X:6760094-6770369:5534-5579
GCGTCCGACTATGAGATGGATGATAATTCAGAGCAAGTGGATACA
>X:6760094-6770369:5606-5649
gacgatgatgcgcttaccgacgattgggtggatctggagactg
>X:6760094-6770369:5660-5705
TCCAAGTCCGCCAAGAGCATTTTTAGAGTGTTCAATCGCTTCTGC
>X:6760094-6770369:5703-5746
gctcgcgtttaaacaaattaccccgatccagcagagcagtgcc
>X:6760094-6770369:5848-5893
GGAGCCAGAGATGGGGGATTCATCCACATCTACAGGTGCTAAGTC
>X:6760094-6770369:5899-5942
cgaacgggtggagaatgtgggctttcaaaagccctcttcagac
>X:6760094-6770369:5974-6017
ctgcgtgcagccgggttgcactttcctcttttccaatgagctg
>X:6760094-6770369:6015-6060

TGGAAGGCCTCGAGAATCATTTTGCGTTAGAGCACCCTCTTGTTC
>X:6760094-6770369:6079-6124
CATGTGCCGTCAGAAAATCACGGCAACGGAAACGAATCTCAGAAT
>X:6760094-6770369:6145-6188
gagggacgtgcacatgaaggacatatccaccctgcctcctcct
>X:6760094-6770369:6198-6241
cggttgaaagcccagccgttattgaatcctgcctgaatcagcg
>X:6760094-6770369:6239-6284
CGTGAACCAGTACCTGAATCAGAACCTGATCCCGTTCCTGAGCTT
>X:6760094-6770369:6312-6355
gggatcgccttgttgtggattcacaagcggaaaagagccaacc
>X:6760094-6770369:6353-6398
CCGGTAGCAATAGTTGTCAGTGATGATGATAATCCGCGAAATGGG
>X:6760094-6770369:6566-6611
ATCGGCCAGGTCCTTAGCAGAACTCAGATTTCAGCTAACTCACGG
>X:6760094-6770369:6658-6701
tgctgccgttgaagagaatcgtaatcgattcaggtgcatggcc
>X:6760094-6770369:6699-6743
CCACCAACTGCAATTTTGTTGCTCACAAGCTCATGTTCATGCGG
>X:6760094-6770369:6815-6860
GCAGTCGATGTGGATGATTACTTGCGCCACGGAGTGATCATTCAC
>X:6760094-6770369:6874-6919
CTCCGAACTGGAGAGTTCAACTGGACCACCATCTGTTACCCAGAA
>X:6760094-6770369:6959-7002
ccaccaccaactcctcaagtcactctgtctgatgtggtcctgg
>X:6760094-6770369:7016-7061
ACCGGATACAGCGGTGAGTGAACTAGACTAATGCTAATATCTTTA
>X:6760094-6770369:7059-7104
TAAATTACTTAGGCTTTCATCTTTATTCCAGAGGATAAACTGTAC
>X:6760094-6770369:7133-7178
GCTGACAGATGAGCAGCTTGTAAACCATTTGCGCTACCACATTCG
>X:6760094-6770369:7310-7354
ATCGGTCAACCAGCGCATAATGATGTATCACATGAGCACGGTGC
>X:6760094-6770369:7404-7449
CCCGTGAAGATCGACGCGAGTAAGAAGAACGTAGAAAGCGAGTTC
>X:6760094-6770369:7459-7502
tcgtggaacagcccctttggcaacctccagatgcaggatttcca
>X:6760094-6770369:7628-7673
CTTCTCCACCACGTCAAGGGTTAACATGCAGATGCACCTCTATGA
>X:6760094-6770369:7700-7745
CTCCAAATTGGCGGACTTGATAGTTCCAGCAACCTCTTCGGTATT
>X:6760094-6770369:7782-7827
CCAGGCAAAGATTCAGAAAAACCATCTACTTCCGGACAAAGTGGT
>X:6760094-6770369:7872-7915
aagcccatcaagccaccgttacgctatgtgcccccggaccaac
>X:6760094-6770369:7920-7963
cgctgtggcttcctccgatgtagcgtcctttgtttttcggaat

>X:6760094-6770369:7971-8016
CGCAAACACATGCAGGCTAACCACAAATACTCGGAGGTGGTAAGG
>X:6760094-6770369:8033-8078
CTGCCAGGGTCAGTTTGGAGTAGATAAGTACTTTGACCATCTTGC
>X:6760094-6770369:8144-8189
GCGGCACATACAGGAACGTCACAATATTCAAGATGTGGACATGAT
>X:6760094-6770369:8195-8238
ccgccataatgacagcaacaaaacgaccgaagcccgctggctg
>X:6760094-6770369:8238-8283
AAGGCGCGTAAGTGTTGAATTTAACTGACCTTCATTTGAAAAAGC
>X:6760094-6770369:8281-8326
GCCCACTCAAGTGTTATATCCCCACTCTTTAGCTAAATTGGCACG
>X:6760094-6770369:8324-8369
CGTCATTCGCTAATGGAGTACACGTGTAACCTGTGCCTCAAGTAC
>X:6760094-6770369:8402-8445
gcgtccgttcacaaacgcaactaccagtaccactgtccgtact
>X:6760094-6770369:8533-8576
CCAGCGCATCGTGTGTAAGAACAAGCAGACGCTAGGCTTCTAC
>X:6760094-6770369:8691-8736
CCGAACGCCAGGTTGTCTTACACATACAAGAGAAGCATCCCCATG
>X:6760094-6770369:8744-8787
ctggcaatggtgcagttcgaacgcgtgcttaatgacatcccga
>X:6760094-6770369:8810-8853
cggcccatcgaagtggagcctgagaaggagatcccgaacaatg
>X:6760094-6770369:8929-8972
GGCGGACGAGTACGGTGAACAAGATGACGCGGTAAGTAAAGTG
>X:6760094-6770369:8974-9019
GCTGATGCAGGCCCAATTTTCAATTTTGTTTTCCTTATGCTCATT
>X:6760094-6770369:9161-9204
tctgctccgagtgcaagagatttaggggcaatgcaaaggcact
>X:6760094-6770369:9274-9318
TGCGCTTACTGCGACTACCGCTATAAAAACAGGCACGATCTTGC
>X:6760094-6770369:9341-9386
ACCTGCCCAATGACCTGAAGCACGTAACAGATGATGAAATTGATG
>X:6760094-6770369:9443-9487
GCGGATTGTGCAGTGTGGTTATGCCAACGAAGGAGACAATTGTC
>X:6760094-6770369:9524-9569
GTTTCTGCTTCCGGCAGCTAGTGTCGCCAGTGATATACCATTGTT
>X:6760094-6770369:9665-9708
CTCAGCCGGGTGGTTTCGATGAGTACATCCAGCACTGCTATAC
>X:6760094-6770369:9732-9777
CTTCCGGGACGTGCACACGTTTAGCGATCTGAAGAGGTACCTTAG
>X:6760094-6770369:9775-9820
AGTCAGGTGCATTACCAATTCCAGAATGGGTTGATTATCACAAAA
>X:6760094-6770369:9818-9863
AAAGCAGTCTCCGTTATACACGTTACAAATCCGACAAATGTATGC
>X:6760094-6770369:9986-10031

TGTCGTTGTTGCGGATCACAAAGCGACGAAAAACGCTTAATCCTG
>X:6760094-6770369:10178-10223
CAACCAGCAACTTGGATCAACACTAACACCACAACATTATTATAA
